# Spatiotemporal dynamics of rhizosphere microbial communities under different mulching methods in spring maize

**DOI:** 10.3389/fpls.2026.1732283

**Published:** 2026-02-04

**Authors:** Jianlong Wu, Kuo Chen, Lianghao Sheng, Haoran Han, Jiaxiu Li, Zhengyu Guo, Shuai Gong, Haoyu Wang, Li Chen, Zhongdong Zhang, Fei Gao

**Affiliations:** 1 College of Agronomy, Shanxi Agricultural University, Taiyuan, China; 2 Institute of Maize, Shanxi Agricultural University, Xinzhou, China

**Keywords:** community structure and composition, maize, plastic film mulching, rhizosphere microbiome, spatiotemporal dynamics

## Abstract

**Introduction:**

Plastic film mulching is a critical practice in arid agroecosystems, yet its spatiotemporal impacts on the rhizosphere microbiome remain poorly understood.

**Methods:**

Here, we investigated how no-mulching (CK), on-film hole sowing (UPM), and film-side planting (FPM) shape the bacterial and fungal communities in the maize rhizosphere across developmental stages (V12 and R6) and soil depths (10, 20, and 30 cm).

**Results:**

Concurrently, both mulching strategies increased maize yield relative to CK, with FPM ultimately outperforming UPM (19.05% vs. 6.24%). Amplicon sequencing showed that mulching strongly structured the rhizosphere microbiome with clear spatiotemporal variation. Bacterial and fungal communities exhibited contrasting patterns: bacteria responded mainly in topsoil at V12 and across all depths by R6, whereas fungi responded across the soil profile at V12, with responses weakening with depth at R6. Mulching—particularly UPM—reduced key taxa, including the nitrifying genus *Nitrospira* and symbiotic *Glomeromycota*. Correlation analyses revealed significant associations between these taxonomic shifts and maize yield components, consistent with Nitrospira’s preference for aerobic conditions. Functional predictions suggested UPM favored communities with higher representation of anaerobic decomposition pathways, whereas FPM supported greater potential for aerobic heterotrophy and nitrogen-related processes.

**Discussion:**

Although microbial shifts were correlated with yield components, yield increases were likely dominated by the direct physical effects of mulching. Overall, distinct mulching strategies generated divergent rhizosphere trajectories, with FPM potentially offering a more sustainable option for dryland maize production.

## Graphical Abstract

**Figure:**
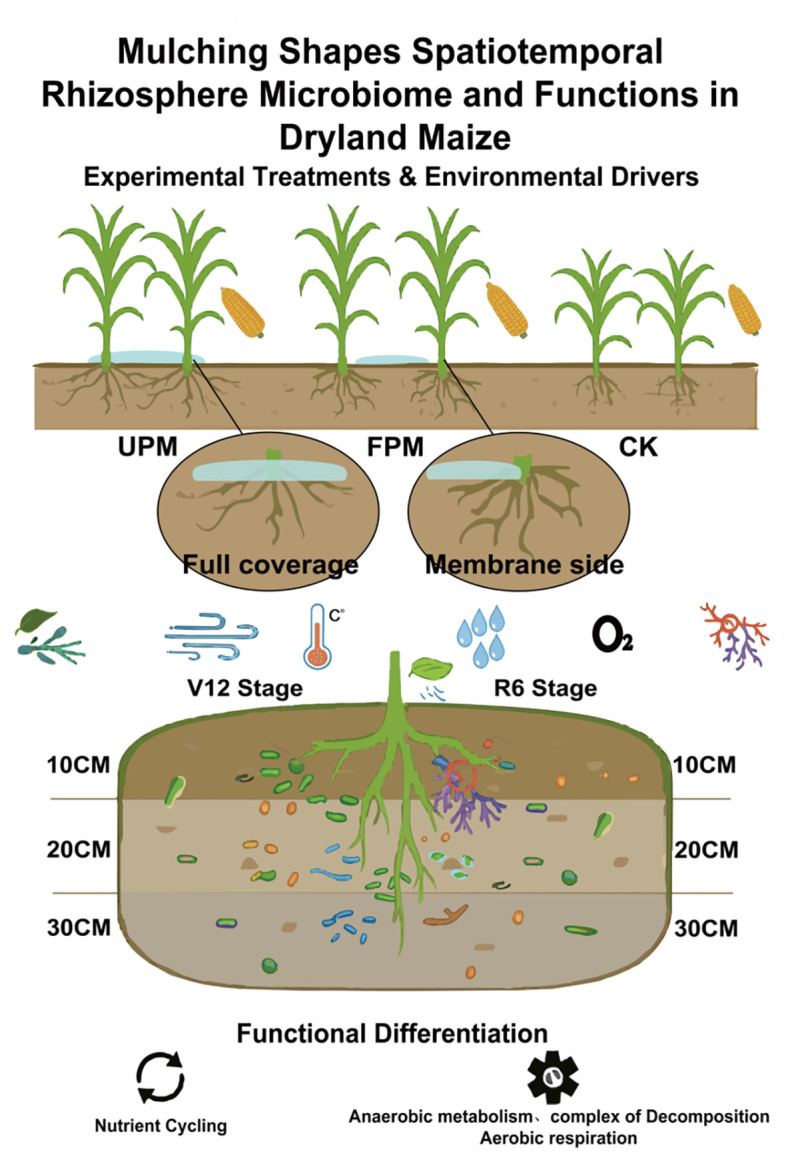

## 1 Introduction

Dryland ecosystems, accounting for approximately 41% of the global terrestrial surface, are critical frontiers for global food security, particularly in regions like northern China (Prăvălie, 2016; Zhang et al., 2017). Maize *(Zea mays* L.) cultivation in these rainfed systems is perennially challenged by water scarcity, making agronomic innovations like plastic film mulching essential for stabilizing and enhancing productivity (Bu et al., 2013; Huang et al., 2024). However, despite these well-documented agronomic benefits, how mulching alters rhizosphere microbial communities—and whether it functions as an environmental filter shaping microbial community structure—remains insufficiently understood. The rhizosphere microbiome, often termed the “plant’s second genome,” is a key determinant of plant health and ecosystem functioning, mediating critical biogeochemical processes such as nutrient cycling, disease suppression, and stress mitigation (Berendsen et al., 2012; Bulgarelli et al., 2015). The composition and structure of this complex community are shaped by a dynamic interaction between soil properties, climatic conditions, and agronomic management practices (Fierer, 2017). Plastic film mulching, by dramatically altering soil temperature, moisture, and aeration, imposes a strong, deterministic selection pressure on the microbial community (Zhu et al., 2018; Tang et al., 2022). However, how this selection pressure varies across different life kingdoms (i.e., bacteria and fungi) and spatiotemporal scales remains a central, unresolved question in microbial ecology. Bacteria and fungi, with their fundamentally different biological and physiological traits—such as unicellular versus filamentous growth forms, distinct cell wall compositions, and divergent nutrient acquisition strategies—are expected to respond divergently to environmental gradients (Bardgett and van der Putten, 2014). Indeed, large-scale studies have confirmed that drivers such as plant diversity can differentially structure bacterial and fungal communities, suggesting they are governed by distinct response patterns (Prober et al., 2015). Yet, few studies have directly compared their responses to agricultural perturbations like mulching within the same experimental framework.

Furthermore, the ecological consequences of these community shifts, particularly for key soil functions like nitrogen (N) and carbon (C) cycling, are far from clear. For instance, while mulching can enhance N uptake by plants, it may simultaneously create anaerobic micro-sites that favor denitrification or suppress critical aerobic processes like nitrification, potentially altering the soil N balance in the long term (Tian et al., 2022; Liu et al., 2024). Understanding how mulching-induced shifts in the abundance of key functional guilds—such as nitrifiers (e.g., Nitrospira), mycorrhizal fungi (e.g., Glomeromycota), and decomposers—translate into changes in the functional potential of the entire microbiome is crucial for evaluating the long-term ecological footprint of this widespread agricultural practice. This is particularly relevant when comparing different application techniques, such as different film-planting patterns, which create distinct micro-niches by varying the degree of soil aeration and root-film contact, and thus may drive different community succession trajectories (Andrew et al., 2012; Yan et al., 2018; Gao et al., 2019).

In light of these knowledge gaps, we conducted a field experiment in a representative dryland agricultural region on the Loess Plateau of China. Using 16S rRNA and ITS amplicon sequencing, we systematically investigated how different film mulching practices shape the spatiotemporal dynamics of rhizosphere bacterial and fungal communities. Specifically, we aimed to test the following hypotheses: (i) mulching acts as a deterministic environmental filter, shaping rhizosphere microbial communities in a spatiotemporally dependent manner. (ii) bacteria and fungi, due to their distinct life strategies, will exhibit divergent community dynamics patterns in response to mulching-induced gradients; and (iii) These structural shifts will drive a significant reorganization of the community's functional potential, particularly in key carbon and nitrogen cycling pathways.

By elucidating the spatiotemporal “fingerprints” of mulching on the rhizosphere microbiome, this study aims to provide a mechanistic, microbial-centric basis for optimizing cultivation strategies to enhance the sustainability and resilience of dryland maize agroecosystems.

## 2 Materials and methods

### 2.1 Study site, experimental setup and sample collection

The field experiment was conducted at the Xinzhou Experimental Station of the China National Maize Industry System (38°24′ N, 112°43′ E), located in a temperate continental monsoon climate zone. The soil at the site was classified as sandy loam. During the maize growing season, the mean air temperature was 20.38°C, and cumulative precipitation was 342.3 mm. Detailed climatic data for the experimental period are provided in **Supplementary Figure S1**.

This study was performed in the fourth year (2024) of a long-term experiment to assess the effects of different mulching practices on the spatiotemporal enrichment of rhizosphere microbes and to identify key microbial taxa. A randomized complete block design (RCBD) was employed, comprising three treatments: (i) film-side planting (FPM) using 60 cm-wide plastic film; (ii) on-film hole-sowing (UPM) using 80 cm-wide plastic film; and (iii) no mulching (CK) as the control. Each treatment was replicated three times, with plot dimensions of 7 m × 5 m (35 m²), resulting in a total of nine plots. The maize cultivar ‘Xianyu 335’ was sown at a plant spacing of 28 cm. A controlled-release compound fertilizer (28:15:8, N:P_2_O_5_:K_2_O) was applied at a rate of 472.5 kg ha^-^¹. Sowing was carried out on 2 May 2024, and the harvest took place on 28 September 2024. Standard local agronomic practices for pest, disease, and weed management were followed throughout the growing season.

Rhizosphere soil samples were collected from maize plants at the V12 (twelve-leaf) and R6 (physiological maturity) growth stages (Chen et al., 2019). These two stages were selected to represent the peak of vegetative growth with vigorous root activity (V12) and the final reproductive stage characterized by root senescence and cumulative treatment effects (R6). In each plot, sampling was conducted at three soil depths (10, 20, and 30 cm) using a standard five-point sampling method. At each sampling point, roots within each specific soil layer were carefully exposed. The plants were carefully uprooted, and loose soil was gently shaken off. The rhizosphere soil tightly adhering to the lateral roots at that specific depth was then collected using a sterile brush. To ensure representativeness and minimize heterogeneity, the rhizosphere soil from these five points at the same depth was thoroughly mixed to form one composite sample per plot. A total of 54 composite samples (3 treatments × 3 replicates × 2 stages × 3 depths) were obtained. From each composite sample, a 2 g subsample was homogenized for subsequent analysis, immediately frozen in liquid nitrogen, and stored at −80°C until further processing.

### 2.2 Yield and plant dry matter accumulation

At harvest, the total number of plants, the number of prolific plants (i.e., those bearing double ears), and the number of lodged plants were recorded for each plot. In each plot, 20 representative ears were randomly collected from the two central rows. These ears were air-dried and transported to the laboratory for yield component analysis. The number of kernels per ear and the thousand-kernel weight (TKW) were subsequently measured. Grain yield of spring maize was calculated and standardized to a 14 % moisture content using the following formula (Guo et al., 2022):


$$Yield(kg\;ha^{-1})=Number\;of\;ears\;per\;unit\;area\;\times \;Kernels\;per\;ear\;\times \;Thousand\;kernel\;weight\;(g)\;/1000\;\times \;(1-actual\;moisture\;content)\;/\;(1-0.14)$$

At the 6-leaf stage (V6), 12-leaf stage (V12), tasseling(VT), milk ripening stage (R3), and mature stage (R6) of the maize, three representative maize plants with consistent growth were randomly selected from each plot. The aboveground part of the plant was sampled from the base of the maize plant near the ground surface, and the stems, leaves, sheaths, bracts, and other organs were packed in sampling paper bags, marked for processing, and placed in an oven. The samples were incubated at 105°C for 30 min to inactivate the enzymes and were then heated to a constant weight at 65°C. The dry samples were weighed and recorded using a balance that was accurate to 0.01 g.

### 2.3 Soil water content and soil temperature measurements

At the V6, V12, VT, R3, and R6 stages of maize development, soil samples were collected from the 0–60 cm soil profile in 20 cm increments using a soil auger. Sampling points were located within the crop row between two adjacent plants. The collected samples were then oven-dried at 105°C to a constant weight to determine the gravimetric soil water content.

Soil temperature was monitored at fixed positions within each plot using bent-stem glass mercury thermometers. On the day of sowing, thermometers were installed in the center of each plot, adjacent to a seed between two maize plants, at depths of 5, 10, and 20 cm. From sowing to harvest, soil temperature readings at each depth were recorded at 5-day intervals at 08:00, 14:00, and 20:00 h. The daily mean soil temperature was calculated as the arithmetic mean of these three readings. For each developmental stage, the temperature difference between the air and the 5–20 cm mean soil temperature was also determined.

### 2.4 Rhizosphere microbial niche breadth

The niche breadth of the rhizosphere microbial community was calculated using Levins’ formula. This index reflects both the number of distinct habitats a species occupies and the evenness of its distribution among them, thereby characterizing the extent to which an organism or population utilizes the available spectrum of resources (Hanski, 1978). All niche breadth calculations were performed in the R environment (version 4.3.1) using the spaa package.

### 2.5 Microbial high-throughput sequencing and analysis

#### 2.5.1 DNA extraction and amplification

Genomic DNA was extracted from rhizosphere soil samples using the MagPure Soil DNA LQ Kit (Magan, China) according to the manufacturer’s protocol. The concentration and purity of the extracted DNA were assessed using a NanoDrop 2000 spectrophotometer (Thermo Fisher Scientific, USA) and verified by agarose gel electrophoresis. DNA samples were then stored at −20°C until further analysis.

PCR amplification was performed using the extracted genomic DNA as a template, barcoded primers, and Takara Ex Taq high-fidelity polymerase (Takara, Japan). For bacterial community analysis, the V3–V4 hypervariable region of the 16S rRNA gene was amplified with the primers 343F (5′-TACGGRAGGCAGCAG-3′) and 798R (5′-AGGGTATCTAATCCT-3′) (Nossa, 2010). For fungal community analysis, the ITS1 variable region of the ITS gene was amplified using the primers ITS1F (5′-CTTGGTCATTTAGAGGAAGTAA-3′) and ITS2 (5′-GCTGCGTTCTTCATCGATGC-3′) (Mukherjee et al., 2014).

#### 2.5.2 Library construction and sequencing

The PCR amplification products were verified by agarose gel electrophoresis and subsequently purified using AMPure XP beads (Beckman Coulter, USA). These purified products then served as templates for a second round of PCR amplification. Amplicons obtained from the second PCR were purified again using magnetic beads, and their concentrations were quantified with a Qubit fluorometer (Thermo Fisher Scientific, USA). Prior to sequencing, all amplicon concentrations were normalized.

Sequencing was performed on an Illumina NovaSeq 6000 platform (Illumina, USA) to generate 250 bp paired-end reads. The sequencing service was provided by OE Biotech Co., Ltd. (Shanghai, China).

#### 2.5.3 Bioinformatics analysis

Bioinformatic processing of the sequencing data was conducted by OE Biotech Co., Ltd. (Shanghai, China). Raw sequencing reads were obtained in FASTQ format. Primer sequences were removed using Cutadapt. The trimmed paired-end reads were processed using the DADA2 plugin within the QIIME 2 pipeline (version 2023.2) (Callahan et al., 2016; Bolyen et al., 2019) to perform quality filtering, denoising, merging, and chimera removal, generating an amplicon sequence variant (ASV) abundance table and representative sequences. Taxonomic classification was carried out using the q2-feature-classifier plugin against the SILVA database (version 138) for bacteria and the UNITE database (version 9.0) for fungi. Alpha diversity indices (ACE and Shannon) were calculated using QIIME 2. Functional prediction was performed using PICRUSt2 and FAPROTAX for bacteria, and FUNGuild for fungi.

#### 2.5.4 Statistical analysis

All statistical analyses were performed in R (version 4.3.2). For alpha diversity indices and soil temperature and moisture, differences among treatments were tested using one-way analysis of variance (ANOVA) followed by Duncan’s multiple range test for normally distributed data, or the Kruskal–Wallis test for non-normally distributed data. Differences in microbial community structure (β-diversity) were assessed using Permutational Multivariate Analysis of Variance (PERMANOVA) based on Binary Jaccard distance matrices with the vegan package. Key microbial taxa were screened using Random Forest analysis implemented in the randomForest package. Correlations between key microbial taxa and environmental factors or maize yield were evaluated using Spearman’s rank correlation analysis. Data visualization was performed using the ggplot2 package. Statistical significance was defined at *P* < 0.05.

## 3 Results

### 3.1 Spring maize yield and rhizosphere environmental conditions

#### 3.1.1 Yield

All yield and yield component data are presented in **Table 1**. Among the three treatments, film-side planting (FPM) achieved the highest grain yield. Specifically, the grain yield under FPM was significantly higher by 10.92% and 17.85% compared to on-film hole sowing (UPM) and the unmulched control (CK), respectively. The yield advantage of FPM was primarily driven by a significant increase in both ear number and kernels per ear. Both mulching treatments (FPM and UPM) significantly increased these two yield components compared to CK. In contrast, the thousand-kernel weight (TKW) did not differ significantly among any of the treatments.

**Table 1:** Effects of different mulching practices on ear characteristics, yield, and yield components of spring maize.

Treatment	Ear L. (cm)	Ear D. (mm)	Ears (ha^-^¹)	KPE	TKW (g)	Yield (kg ha^-^¹)
CK	15.4b	4.6b	78045.0b	447.4b	359.8a	10804.4c
UPM	16.4a	4.8a	75263.6c	496.9ab	356.9a	11478.9b
FPM	16.4a	4.7ab	80910.2a	516.3a	354.4a	12732.0a

CK, no mulching; UPM, on-film hole-sowing; FPM, film-side planting; Ear L., ear length; Ear D., ear diameter; KPE, kernels per ear; TKW, thousand-kernel weight. Different letters indicate significant differences among treatments (*P* < 0.05).

#### 3.1.2 Effects of different mulching practices on soil moisture and temperature

Compared with the unmulched control (CK), both film mulching treatments significantly improved soil hydrothermal conditions (**Supplementary Figure S2**). However, the two mulching strategies exhibited distinct advantages. The on-film hole sowing (UPM) treatment consistently resulted in the greatest increase in soil temperature, particularly during the early growth stages. In contrast, the film-side planting (FPM) treatment showed the most pronounced water-conservation effect, maintaining a significantly higher average soil water content throughout the growing season compared to both UPM and CK (**Supplementary Figure S2**).

#### 3.1.3 Dynamics of dry matter accumulation under different mulching practices

**Figure 1:**
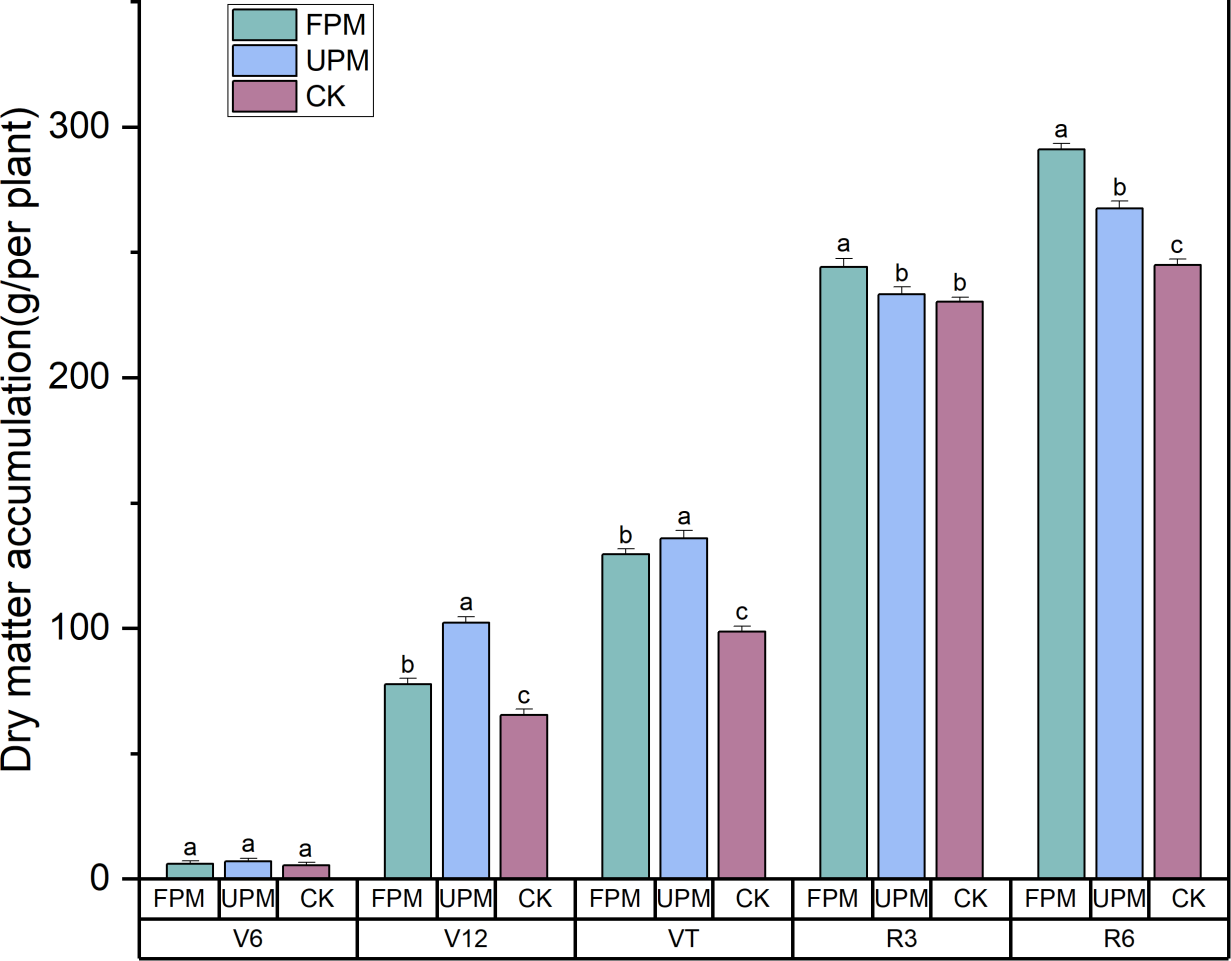
Dynamic changes in maize dry matter accumulation under different mulching treatments. FPM, film-side planting; UPM, on-film hole sowing; CK, no mulching. Data are presented as means ± standard error (n = 3). Different lowercase letters above the bars within the same growth stage indicate significant differences among treatments at P < 0.05 according to Tukey's HSD test.

To assess the dynamic effects of different mulching practices on biomass accumulation, we measured the dry matter of individual plants at five key growth stages (**Figure 1**). Film mulching significantly promoted dry matter accumulation, but the effects were stage-dependent. During the rapid vegetative growth phase (V12 to VT), both mulching treatments surpassed the CK control, and the UPM treatment was significantly superior to FPM. However, this trend reversed during the reproductive stages. By the R3 stage, FPM’s dry matter accumulation had already surpassed that of UPM, and at the final R6 stage, the overall biomass accumulation followed a clear pattern of FPM > UPM > CK.

### 3.2 Community composition and structure

Sequencing of the 16S rRNA and ITS amplicons yielded a total of 15,515 and 17,614 bacterial amplicon sequence variants (ASVs) and 1,988 and 3,009 fungal ASVs from samples at the V12 and R6 stages, respectively. The species accumulation curves indicated that the sequencing depth was sufficient to capture the majority of microbial taxa present in the samples (**Supplementary Figure S3**).

At the phylum level, the rhizosphere microbiome was dominated by a few key phyla, whose relative abundances showed pronounced spatiotemporal dynamics (**Supplementary Figure S4**). For bacteria, Proteobacteria and Actinobacteriota exhibited opposing depth preferences that shifted between growth stages. For fungi, a striking finding was the exceptionally high relative abundance of Basidiomycota in the mid-depth soil under UPM at V12, while both Ascomycota and Basidiomycota were co-enriched in the deep soil under UPM by R6. Notably, the symbiotic phylum Glomeromycota remained at a consistently low abundance across all mulched conditions.

**Figure 2:**
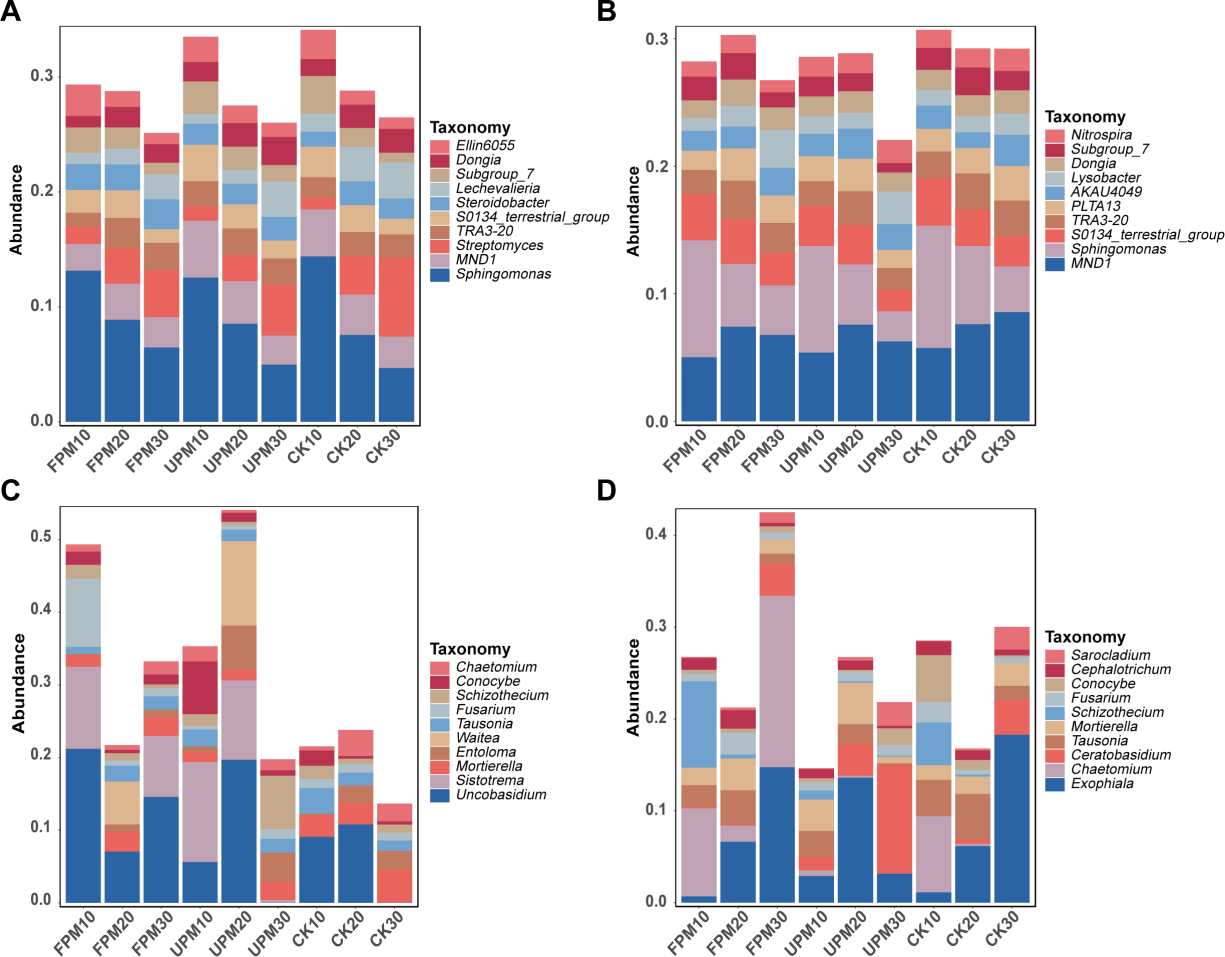
Relative abundance of microbial communities at the genus level across different treatments and developmental stages. **(A, B)** The ten most abundant genera in the bacterial community at the V12 and R6 developmental stages, respectively. **(C, D)** The ten most abundant genera in the fungal community at the V12 and R6 developmental stages, respectively. The x-axis represents the different combinations of treatments and soil depths (10 cm, 20 cm, and 30 cm). The y-axis indicates the relative abundance (%). The legend lists the top ten most abundant genera; all other less abundant genera are grouped as 'Others'.

At the genus level, the microbiome exhibited more fine-grained and functionally relevant shifts in response to treatments (**Figure 2**). During the V12 stage, key bacterial genera displayed distinct niche preferences (**Figure 2A**). *Sphingomonas*, a dominant genus with relative abundances ranging from 4.97% to 14.38%, clearly favored the shallower soil layers under mulching treatments. In stark contrast, the beneficial genus *Streptomyces* was most abundant in the deep, unmulched soil (CK at 30 cm), reaching a peak of 6.87%. This bacterial composition evolved by the R6 stage (**Figure 2B**). A functionally critical shift was the marked suppression of the nitrifying genus *Nitrospira* in the deep soil under both mulching treatments compared to the CK control, where it reached its highest relative abundance.

The fungal community at the genus level was also highly dynamic and treatment-sensitive. At the V12 stage, several genera showed highly localized enrichment, most notably the potential plant pathogen *Fusarium*, whose relative abundance was significantly elevated to 9.42% only under FPM in the topsoil (**Figure 2C**). By the R6 stage, the community structure continued to diverge (**Figure 2D**). *Chaetomium* was exclusively enriched in the deep soil of the FPM treatment, reaching 18.67%. The most remarkable trend was observed for *Ceratobasidium*, which displayed a dramatic increase in relative abundance with soil depth specifically under the UPM treatment, rising from 1.48% at 10 cm to a dominant 11.89% at 30 cm.

### 3.3 Microbial community diversity

#### 3.3.1 Spatiotemporal shifts in microbial diversity and community structure

The impact on α-diversity was primarily observed in species richness (ACE index), whereas the Shannon diversity index, which accounts for both richness and evenness, remained relatively stable (**Supplementary Figures S5**-**S8**). For bacteria, the UPM treatment transiently promoted richness in the topsoil (10 cm) at the V12 stage, but this effect reversed by the R6 stage, where richness was constrained in the deep soil (30 cm). Fungal richness at R6 was also significantly lower in the deep soil under both mulching treatments.

In contrast, the impact on community structure (β-diversity) was more profound and systematic. Permutational Multivariate Analysis of Variance (PERMANOVA) revealed significant and highly spatiotemporally specific effects on both bacterial and fungal communities (**Table 2**). For bacteria, structural differentiation was confined to the topsoil (10 cm) at the V12 stage (R² = 0.17, *P* = 0.013) but expanded to all soil depths by the R6 stage, with the strongest effect size observed in the deep soil (30 cm, R² = 0.17, *P* = 0.001). The fungal community exhibited a divergent spatiotemporal pattern: at the V12 stage, the treatment effect size (R²) increased with depth, peaking at 20 cm and 30 cm (R² ≈ 0.18), whereas by R6, this trend inverted, with the effect size attenuating in the deeper soil layers (**Table 2**).

**Table 2:** Permutational multivariate analysis of variance (PERMANOVA) of rhizosphere bacterial and fungal community structures.

Microbial group	Developmental stage	Soil depth	R²	*P*
Bacteria	V12	10cm	0.165	0.013*
		20cm	0.149	0.210
		30cm	0.155	0.097
	R6	10cm	0.153	0.033*
		20cm	0.160	0.033*
		30cm	0.174	0.001***
Fungi	V12	10cm	0.162	0.024*
		20cm	0.178	0.006**
		30cm	0.177	0.002**
	R6	10cm	0.165	0.001***
		20cm	0.165	0.005**
		30cm	0.161	0.012*

The analysis was based on binary Jaccard dissimilarity matrices for each subset of data. P-values were obtained from 999 permutations. Asterisks indicate the level of significance: *, P < 0.05; **, P < 0.01; ***, P < 0.001.

#### 3.3.2 Rhizosphere microbial niche breadth

**Figure 3:**
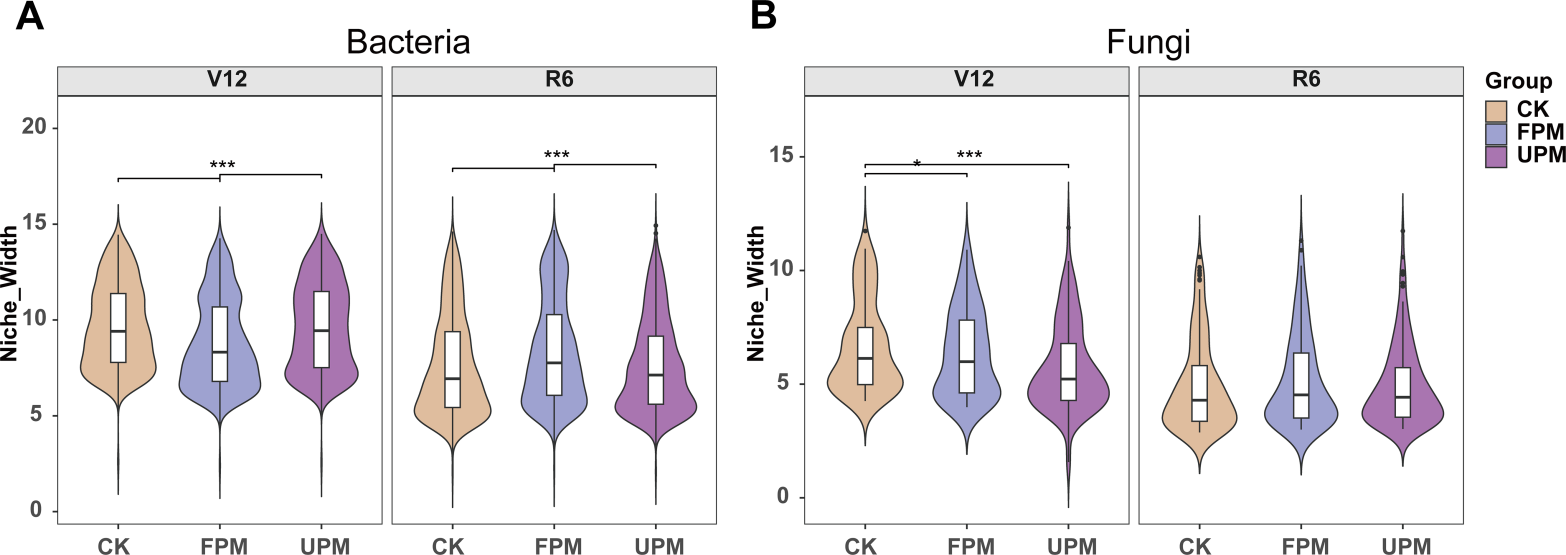
Spatiotemporal changes in the niche width of rhizosphere **(A)** bacterial and **(B)** fungal communities under different mulching treatments. CK: no mulching; FPM: film-side planting; UPM: on-film hole sowing. The violin plots illustrate the probability density of the data, with the internal box plots showing the median and interquartile range. Asterisks above the brackets indicate significant differences between treatments (***, P < 0.001).

Microbial niche breadth exhibited significant and divergent spatiotemporal dynamics between bacteria and fungi (**Figure 3**). A general trend observed was a marked decline in niche breadth for both communities from the V12 to the R6 stage. More importantly, the bacterial community displayed a striking stage-dependent reversal in its response to mulching. At the V12 stage, niche breadth was significantly wider under the CK and UPM treatments compared to FPM. Conversely, at the R6 stage, this pattern inverted, with FPM exhibiting significantly wider niche breadth than both CK and UPM. The fungal community responded differently: its niche breadth was highest under CK at the V12 stage, but by the R6 stage, all significant differences among treatments had disappeared.

### 3.4 Functional prediction of microbial communities

#### 3.4.1 Effects of mulching practices on bacterial functional pathways

**Figure 4:**
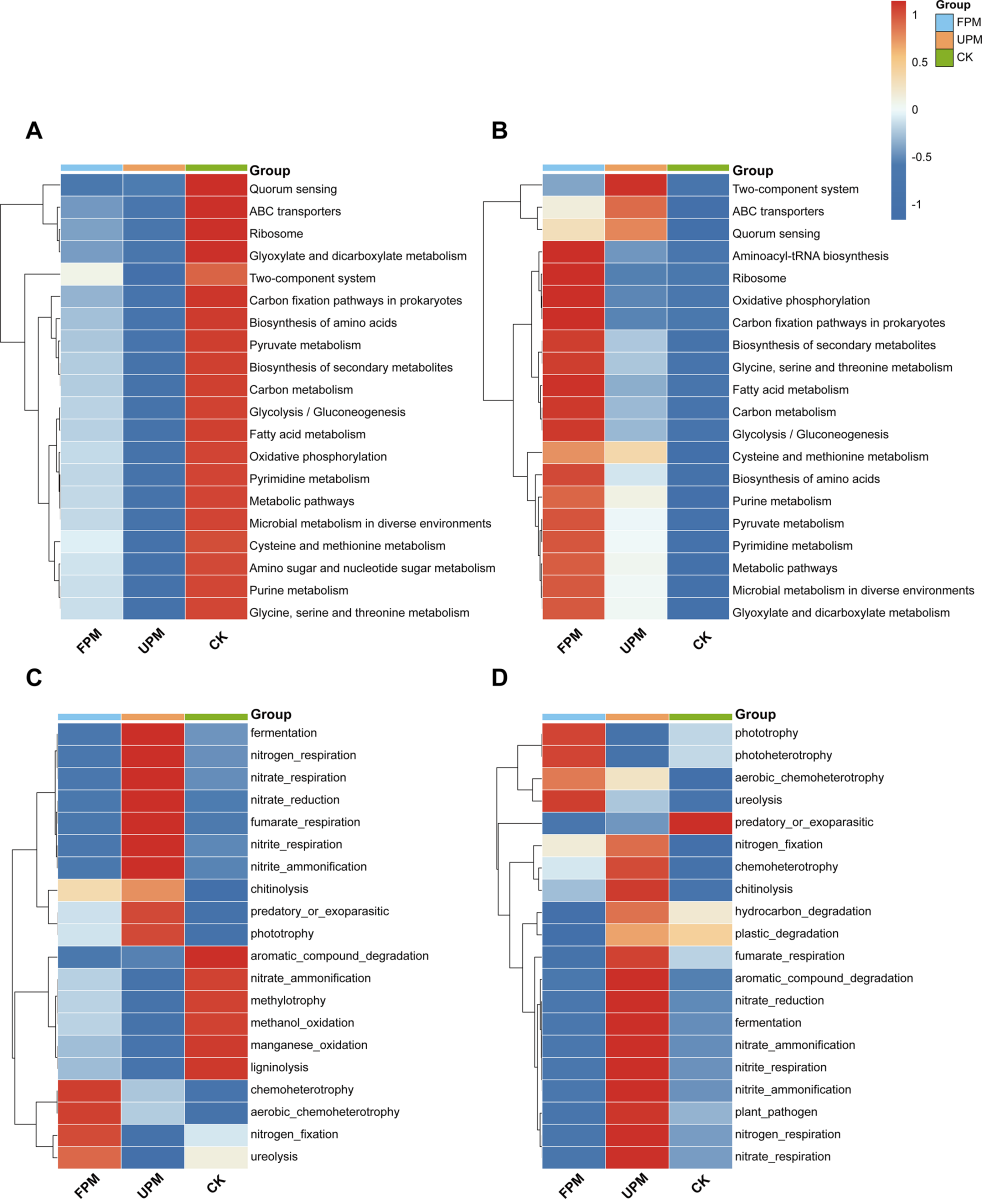
Predicted functional profiles of the soil microbial community based on PICRUSt2 and FAPROTAX analyses.**(A, B)** Heatmaps showing the top 20 most abundant metabolic pathways (KEGG Level 3) in the bacterial community at the V12 and R6 stages, respectively, based on PICRUSt2 predictions. **(C, D)** Heatmaps showing the top 20 most abundant biogeochemical functions in the bacterial community at the V12 and R6 stages, respectively, based on FAPROTAX predictions. In the heatmaps, each row represents a specific function, and each column represents a treatment group. The color intensity reflects the relative abundance of each function (Z-score normalized), with red indicating higher abundance and blue indicating lower abundance. Both rows (functions) and columns (treatments) are hierarchically clustered to visualize their similarities.

Functional prediction using PICRUSt2 revealed that film mulching significantly reshaped the metabolic potential of the rhizosphere bacterial community, with distinct functional profiles observed at the V12 and R6 stages (**Figure 4**, **Supplementary Figure S9**).

A striking temporal shift was observed in the overall metabolic potential. At the V12 stage, the unmulched CK treatment generally maintained the highest potential across the top 20 most abundant metabolic pathways, suggesting a suppressive effect of early-season mulching on core bacterial metabolism (**Figure 4A**). However, this trend completely reversed by the R6 stage. The FPM treatment then exhibited the highest predicted abundances for a wide range of pathways, particularly those related to core metabolism (e.g., Carbon metabolism, Glycolysis), macromolecule biosynthesis (e.g., Ribosome, Biosynthesis of amino acids), and secondary metabolite production (**Figure 4B**).

In contrast, the UPM treatment consistently fostered a community with different functional traits. Throughout both stages, it was associated with pathways related to environmental sensing, stress response, and intercellular communication (e.g., Quorum sensing, Two-component system). At the R6 stage, this was coupled with an enrichment of pathways linked to pathogenicity (e.g., Amoebiasis), suggesting a shift towards a more competitive and potentially antagonistic microbial ecosystem under long-term full mulching (**Supplementary Figure S9**).

#### 3.4.2 Effects of mulching practices on bacterial functional categories

FAPROTAX functional prediction further clarified the distinct ecological roles cultivated by the different mulching practices, consistently showing that UPM promoted anaerobic metabolic functions while FPM favored aerobic processes (**Figure 4**).

During the V12 stage, these functional divergences were already pronounced (**Figure 4C**). The UPM treatment significantly enriched a suite of anaerobic functions, particularly those involved in the nitrogen cycle under hypoxic conditions (e.g., nitrogen respiration, nitrate respiration). In contrast, the FPM treatment fostered a community dominated by aerobic chemoheterotrophs and showed the highest predicted potential for nitrogen fixation and ureolysis. The unmulched CK treatment maintained a unique profile, with the highest potential for degrading complex organic matter. By the R6 stage, this core anaerobic/aerobic division persisted, though some roles evolved (**Figure 4D**). The UPM treatment continued to harbor the highest potential for most anaerobic functions and also became the primary site for the decomposition of specific organic substrates. Notably, the potential for plant pathogenesis was also highest under UPM at this later stage. In a significant shift, the highest potential for nitrogen fixation now occurred under UPM, likely reflecting a switch to anaerobic N-fixing pathways. The FPM treatment, meanwhile, remained a hub for key aerobic processes.

#### 3.4.3 Effects of mulching practices on fungal trophic functions

**Figure 5:**
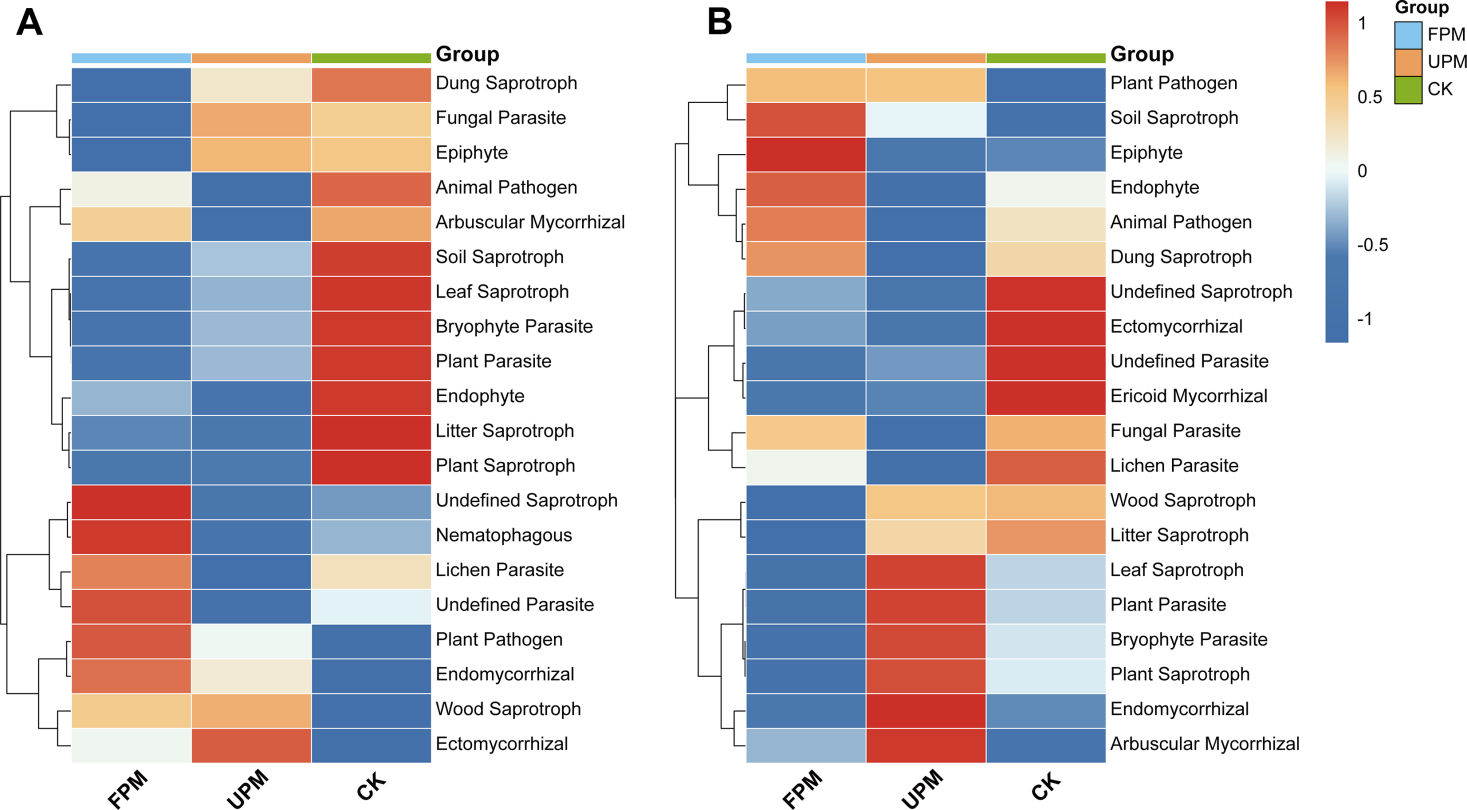
Predicted ecological functions of the soil fungal community based on FUNGuild. **(A, B)** Heatmaps showing the relative abundance of the top 20 fungal trophic guilds at the V12 and R6 developmental stages, respectively. In the heatmaps, each row represents a specific ecological role (guild), and each column represents a treatment group. The color intensity reflects the relative abundance of each guild (Z-score normalized), with red indicating higher abundance and blue indicating lower abundance. Hierarchical clustering of both rows (guilds) and columns (treatments) illustrates the similarities between them.

FUNGuild functional prediction revealed that the different mulching treatments fostered distinct fungal trophic guilds, with these ecological roles shifting significantly between the V12 and R6 developmental stages (**Figure 5**).

During the early V12 stage, the treatments cultivated specialized guilds (**Figure 5A**). The FPM treatment was uniquely characterized by higher abundances of beneficial guilds, including symbiotic (Arbuscular and Endomycorrhizal) and predatory (Nematophagous) fungi. In contrast, the UPM treatment was distinguished by its enrichment of parasitic (Fungal Parasite) and epiphytic guilds. The unmulched CK treatment served as a reservoir for a broader diversity of saprotrophs. By the later R6 stage, a functional role reversal was observed, with the UPM treatment becoming a hub of high fungal activity (**Figure 5B**). It significantly enhanced the potential for a broad spectrum of guilds, most notably saprotrophs (Leaf, Plant, Wood, and Litter Saprotrophs), but also plant parasites and key symbionts (Arbuscular and Endomycorrhizal fungi). The FPM treatment, meanwhile, was primarily enriched in guilds associated with plant surfaces and tissues, such as Epiphyte and Endophyte.

### 3.5 Linking key microbial taxa to ecological functions and maize productivity

To elucidate the potential ecological consequences of the observed community shifts, we first identified the most influential microbial genera driving these changes using Random Forest models (**Supplementary Figures S10**, **S11**). We then performed a series of Spearman's correlation analyses using these key discriminant genera. First, we aimed to infer their functional roles by correlating their abundances with predicted functions (FAPROTAX and FUNGuild) (**Figures 4**, **5**), and then directly linked them to maize productivity indicators to assess their relevance to crop performance.

The analysis identified associations between key taxa and their inferred ecological roles (**Supplementary Figures S12**, **S13**). For bacteria, the most notable finding was the strong positive correlation between Nitrospira and aerobic pathways, alongside its negative correlation with anaerobic N-cycling pathways. This aligns with its known role as an aerobic nitrifier and provides a functional rationale for its suppression under the likely hypoxic conditions of mulched soils. For fungi, the analysis supported the trophic modes of several key genera. For instance, the symbiotic role of *Glomus* was consistent with its strong positive correlation with the Arbuscular Mycorrhizal guild, while the abundance of *Ceratobasidium* at R6 was strongly and positively correlated with the Endomycorrhizal guild, suggesting a specific symbiotic strategy for this genus.

**Figure 6:**
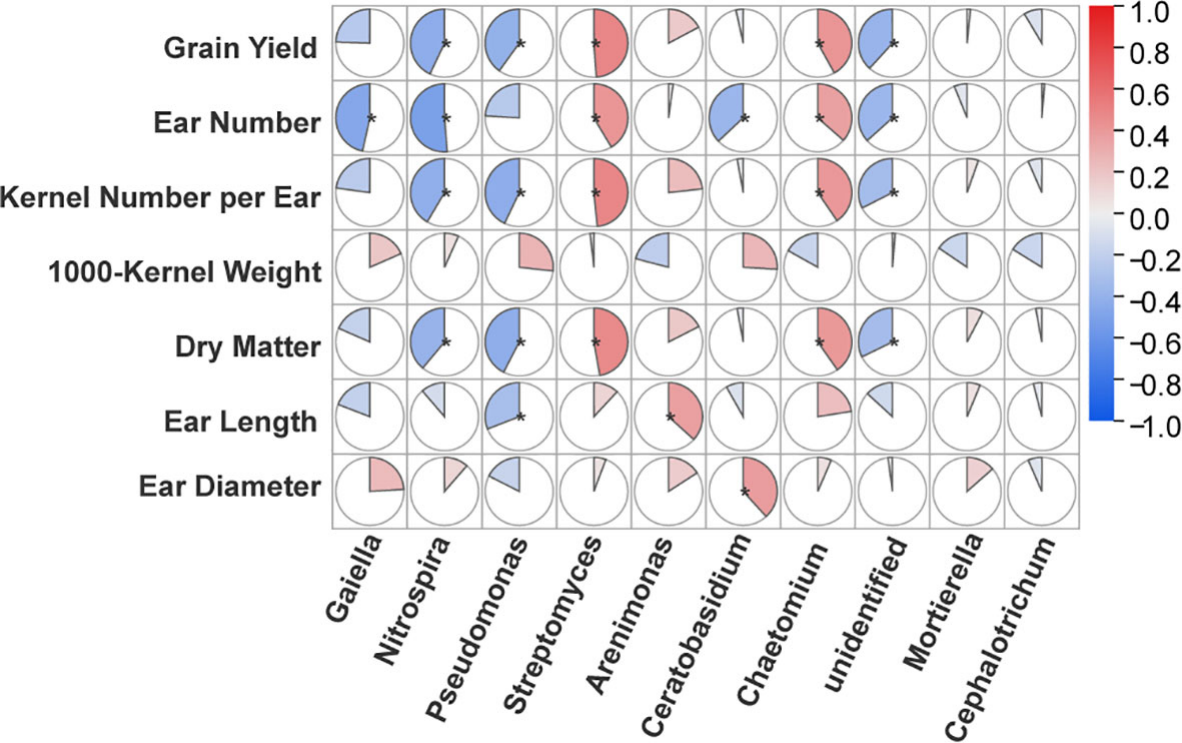
The heatmap displays the Spearman's correlation coefficient (ρ) between maize productivity indicators and the relative abundances of key microbial taxa at the R6 stage. The color scale indicates the direction and strength of the correlation. Asterisks within the cells indicate the level of significance: *P < 0.05; Blank cells indicate non-significant correlations (P ≥ 0.05).

To directly link these microbial shifts to crop performance, we performed a correlation analysis between key microbial taxa and maize productivity indicators (**Figure 6**). A striking pattern of negative correlations was observed, where the relative abundances of the nitrifying genus *Nitrospira* and the dominant genus *Pseudomonas* were significantly and negatively correlated with Grain Yield, Ear Number, and Dry Matter. In contrast, a suite of other key taxa exhibited significant positive correlations with productivity. The beneficial bacterial genus *Streptomyces* was positively correlated with Grain Yield, Kernel Number per Ear, and Dry Matter. Similarly, the fungal genus *Chaetomium* showed strong positive correlations with multiple yield components, including Grain Yield and Ear Number. These findings provide direct, albeit correlational, evidence that the mulching-induced shifts in specific microbial taxa—particularly those with important inferred functions in nutrient cycling and plant health—are closely linked to the final crop performance.

## 4 Discussion

### 4.1 Coordinated regulation of maize growth and the soil microenvironment by film mulching

Agricultural ecosystems in arid and semi-arid regions face the dual challenges of water scarcity and high evaporative demand, which severely constrain crop productivity and resource-use efficiency (Zhang et al., 2022). Film mulching, a well-established agronomic practice, has been widely adopted to mitigate these constraints by improving the soil microenvironment, enhancing water-use efficiency (WUE), and suppressing weed growth (Gu et al., 2018; Xu et al., 2018). Consistent with these established benefits, both the FPM and UPM treatments significantly increased spring maize yield, accompanied by a higher number of effective ears. This result aligns with previous reports on the yield-enhancing effects of mulching in similar dryland regions (Zhang et al., 2022).

The underlying mechanism for this yield improvement is closely linked to the mulching-driven regulation of the soil hydrothermal environment. It is well-documented that during the early seedling stage, film mulching effectively reduces soil evaporation, elevates soil temperature, and improves water retention (Gao et al., 2014). These favorable conditions promote robust early root development and biomass accumulation, laying the foundation for higher final yields. Indeed, the UPM treatment accelerated biomass accumulation during the early to mid-vegetative stages (V12 and VT, likely due to its superior heat-trapping and moisture-preserving capabilities which create an “incubator effect” for early growth (Boatwright et al., 1976). However, this early advantage did not translate to the highest final biomass. Instead, the FPM treatment, despite a slower start, surpassed UPM in dry matter accumulation during the reproductive stages (R3 and R6). This suggests a critical trade-off: the aggressive early growth under UPM may lead to premature senescence, a phenomenon often linked to supra-optimal soil temperatures and hypoxic root conditions under full film coverage (Xu et al., 2015). High soil temperatures, particularly during the reproductive stage, can accelerate plant development but also impair root activity and nutrient uptake, leading to a shortened grain-filling period and reduced final biomass (Li et al., 2013). In contrast, FPM likely achieved this balance by facilitating gas exchange and moderating excessive soil heating. This strategy sustained plant vigor through to maturity, driving the highest final biomass and yield. These findings suggest that the advantage of FPM lies in mitigating late-season physiological stress, rather than merely maximizing early growth.

### 4.2 Spatiotemporal responses of rhizosphere microbial community structure and functional guilds to film mulching

Film mulching fundamentally reshapes the rhizosphere microbiome by altering soil hydrothermal and aeration conditions, leading to pronounced spatiotemporal heterogeneity in community diversity, structure, and composition (Folman et al., 2001; Wang et al., 2021). We acknowledge that microbial community composition is also influenced by soil chemical properties (e.g., pH and organic matter), which were not systematically quantified in the present study. In dryland ecosystems, however, soil temperature and moisture constitute key environmental constraints, and the mulching-induced hydrothermal gradients captured here are therefore considered likely major drivers of the observed microbial patterns, while chemical co-variates may play a contributory role.

Our study revealed that the impact of mulching on microbial α-diversity was subtle and highly context-dependent, a finding consistent with some previous reports (Sun et al., 2021). For instance, at the V12 stage, the UPM treatment transiently promoted bacterial richness in the topsoil, likely due to early improvements in hydrothermal conditions. However, by the R6 stage, this effect diminished, and greater bacterial diversity was observed in the deep soil of the unmulched control, possibly because prolonged mulching created less favorable conditions (e.g., reduced aeration) that constrained diversity (Li et al., 2020).

Unlike the nuanced changes in α-diversity, β-diversity revealed pronounced, treatment-specific shifts in community structure. This disparity underscores that mulching primarily reshapes the identities and relative abundances of microbial taxa, rather than merely altering species richness (Lozupone and Knight, 2007). Initially, treatment effects were restricted to the topsoil. By the R6 stage, however, these structural divergences had propagated into the subsoil. This shift was particularly pronounced for bacteria, with the strongest differentiation occurring at 30 cm (*P* = 0.001). This pattern likely reflects a lagged response of the deep-soil microbiome to the cumulative alteration of soil conditions and the changing inputs from senescing roots. Conversely, the fungal community exhibited an attenuating response pattern, with treatment effects diminishing in deeper soil layers at the later stage. This marked divergence from bacteria underscores that these two kingdoms employ distinct strategies to navigate mulching-induced gradients. Bacteria, as rapid-responding r-strategists, likely track localized physicochemical shifts. In contrast, fungi may buffer against deep-soil heterogeneity through their extensive mycelial networks, allowing them to integrate environmental signals across larger spatial scales and maintain greater stability in the subsoil (Bahram et al., 2018).

Consistent with previous studies on gramineous crops, the maize rhizosphere was dominated by Proteobacteria, Actinobacteriota, Ascomycota, and Basidiomycota (Peiffer et al., 2013; Coleman-Derr et al., 2016). More importantly, the results revealed dynamic responses of key functional taxa, exemplified by the nitrifying genus *Nitrospira*, which was markedly suppressed in deeper soil layers under mulched treatments; this pattern in likely microaerobic soil layers at the R6 stage strongly points to oxygen limitation as a key driver, a finding that contrasts with hypotheses emphasizing substrate availability as the primary control in some agricultural systems (Zhang et al., 2012). This suggests that under long-term film mulching, the physical barrier to gas exchange may override the biochemical benefits of enhanced mineralization, fundamentally altering the nitrogen cycle. Similarly, the consistent suppression of the symbiotic arbuscular mycorrhizal fungi (*Glomeromycota*) under both mulching treatments is a critical finding, indicating a potential ecological trade-off where the physical benefits of mulching (e.g., improved water status) may come at the cost of weakening a key plant-microbe mutualism (Treseder, 2004). The sustained suppression of *Glomeromycota* observed in this study suggests potential longer-term ecological implications, as AMF contribute to phosphorus acquisition and soil aggregation through glomalin production (Rillig, 2004). However, these implications cannot be directly inferred from the present short-term observations, and long-term studies are needed to evaluate whether management strategies, such as crop rotation or organic amendments, can mitigate these effects. Furthermore, the enrichment of the potential plant pathogen *Fusarium* under FPM at the V12 stage, and the accumulation of the root-associated genus *Ceratobasidium* under UPM at the R6 stage, further illustrate how specific mulching regimes create distinct ecological niches that favor different functional guilds.

### 4.3 Predicted functional potential of rhizosphere microbiomes and inferred ecological mechanisms

Shifts in community structure ultimately translate into a reconfiguration of the rhizosphere microbiome’s functional potential. While amplicon sequencing primarily reveals community structure, function prediction based on marker genes can still provide critical hypotheses and directions for understanding how mulching impacts the potential functions of the ecosystem (Ling et al., 2022). While we acknowledge that marker-gene-based predictions are inferential, the functional profiles derived from PICRUSt2, FAPROTAX, and FUNGuild demonstrated remarkable consistency with our taxonomic observations and environmental data. This multi-evidence convergence robustly indicates a systematic reconfiguration of rhizosphere carbon and nitrogen cycling and pathogen dynamics under film mulching.

Our study indicates that the two mulching strategies appeared to foster fundamentally different rhizosphere ecosystems. During the early V12 stage, the UPM treatment created a high-temperature, high-moisture, and poorly aerated microenvironment that strongly selected for anaerobic metabolic guilds, particularly those involved in denitrification (Yu et al., 2021). We hypothesize that this hypoxic stress imposed physiological burdens on the root system, potentially contributing to the premature senescence often observed in on-film sowing. In contrast, the FPM treatment, likely by providing superior gas permeability, established a more balanced rhizosphere dominated by aerobic heterotrophs and nitrogen-fixing bacteria. Meanwhile, the unmulched CK treatment represented a baseline state, supporting a community focused on degrading complex native organic matter. By the R6 stage, the primary driver of functional differentiation shifted from purely environmental selection to a complex interplay between environmental stress and substrate availability from senescing roots (Gunina and Kuzyakov, 2015). Under UPM, the abundance of plant residues fueled a massive enhancement of anaerobic metabolism and decomposition pathways, but also increased the potential for plant pathogens. The microbial community under UPM was characterized by the enrichment of pathways associated with quorum sensing and environmental adaptation, indicating a community with intensified microbial interactions under relatively favorable resource conditions. In contrast, the FPM treatment was associated with higher predicted functional potentials in core metabolic pathways, including carbon metabolism, oxidative phosphorylation, and macromolecule biosynthesis. These patterns are consistent with a microbial community exhibiting enhanced metabolic capacity under FPM, although such inferences are based on functional predictions rather than direct measurements of microbial activity.

The fungal community displayed parallel, yet distinct, functional trajectories. At the V12 stage, the FPM treatment selectively promoted beneficial guilds, including symbiotic AMF and predatory nematophagous fungi, indicating the potential establishment of a disease-suppressive rhizosphere. The UPM treatment, in contrast, tended to enrich fungal parasites and epiphytes. As plant senescence progressed to the R6 stage, the UPM treatment became a hotbed for both saprotrophic and pathogenic fungi, likely fueled by nutrients from decaying roots. Intriguingly, it also harbored the highest potential for AMF at this stage, suggesting a complex trade-off between pathogenic and symbiotic interactions under these specific conditions. It is noteworthy that while different prediction tools pointed to different treatments as having higher pathogenic potential, the overarching conclusion is that the warm, moist environment created by any form of mulching can increase the risk of proliferation for certain thermophilic or hygrophilous pathogens.

### 4.4 Linking rhizosphere microbiome shifts to ecological functions and maize productivity

A key observation was the significant correlation between specific microbial taxa and maize productivity. A striking, albeit counter-intuitive, observation was the significant negative correlation between the relative abundances of the nitrifying genus *Nitrospira* and the versatile genus *Pseudomonas* with multiple yield components, including grain yield and dry matter. However, we argue that this does not necessarily imply a direct suppressive effect of these microbes on plant growth. Instead, this correlation likely reflects their role as bio-indicators of different ecosystem states. It is well-established that at larger scales, microbial community structure is predominantly governed by abiotic factors (Fierer and Jackson, 2006). In our system, *Nitrospira*, a known aerobe, thrived in the unmulched (CK) treatment, which, due to water and heat stress, was also the lowest-yielding system. Thus, the high abundance of *Nitrospira* is an indicator of the well-aerated but less productive CK environment, rather than a direct cause of low yield. This underscores the primacy of physicochemical drivers in our arid system, where the powerful water- and heat-conserving effects of mulching are the dominant determinants of yield, potentially overriding the subtler effects of individual microbial taxa. Conversely, the significant positive correlations between the beneficial genus *Streptomyces* and the fungal genus *Chaetomium* with grain yield and other productivity indicators provide compelling, albeit correlational, evidence for their contribution to a high-yielding rhizosphere. These taxa were more abundant in the FPM treatment, which ultimately produced the highest yield. This suggests that the functionally balanced, aerobic microbial community fostered by FPM is a key feature of a high-productivity agroecosystem.

To further explore the functional underpinnings of these microbe-yield relationships, we correlated these key taxa with their predicted ecological functions (detailed in **Supplementary Figures S12**, **Supplementary Figures S13**). This analysis provided strong support for their inferred roles. For instance, the positive correlation of *Nitrospira* with aerobic chemoheterotrophy and its negative correlation with anaerobic N-cycling pathways confirmed its aerobic nature, reinforcing the hypothesis that its suppression under mulching is driven by oxygen limitation (Daims et al., 2015). Similarly, the symbiotic role of *Glomus* (Berruti et al., 2016) and the multi-trophic nature of *Mycosphaerella* (Crous et al., 2009) were also consistent with their correlations to respective functional guilds. A particularly important finding was the strong positive correlation of *Ceratobasidium* with the Endomycorrhizal guild, suggesting its potential role as a key root-associated symbiont under late-season UPM conditions (Weiß et al., 2011). These taxon-function linkages, while inferential, provide a mechanistic basis for understanding how the specific microbial players shaped by mulching could contribute to the overall functional trajectory of the rhizosphere and, ultimately, its association with crop productivity.

## 5 Conclusion

Different mulching practices reshaped the rhizosphere microbial community of dryland spring maize in a pronounced spatiotemporal manner. Film mulching altered both the composition and structure of bacterial and fungal communities, with divergent spatiotemporal patterns across growth stages and soil depths. These changes were accompanied by a reduced niche breadth and the suppression of beneficial taxa, including Glomeromycota, indicating that mulching-induced hydrothermal conditions impose stronger environmental filtering on the soil microbiome. Microbial community shifts were significantly correlated with maize yield components. However, yield enhancement under mulching was likely dominated by improved soil water and heat conservation, while microbial changes may have played a secondary or indicative role in this field-scale system. The ecological implications of these shifts remain unresolved. In particular, the long-term suppression of functional guilds such as nitrifiers and symbiotic fungi under sustained mulching warrants further investigation, especially with respect to soil health and potential dependence on external inputs. The spatiotemporal patterns identified here provide a framework for future studies aimed at reconciling short-term yield improvement with long-term agroecosystem sustainability.

## Data Availability

The datasets presented in this study can be found in online repositories. The names of the repository/repositories and accession number(s) can be found below: https://www.ncbi.nlm.nih.gov/, BioProject ID:PRJNA1348696.
