# Supplementary material for: Spatiotemporal dynamics of rhizosphere microbial communities under different mulching methods in spring maize
Source: Front Plant Sci. 2026 Feb 4;17:1732283. doi: 10.3389/fpls.2026.1732283 (PMC12913508; doi:10.3389/fpls.2026.1732283)
Supplement: Supplementary file 1 [file DataSheet1.docx]

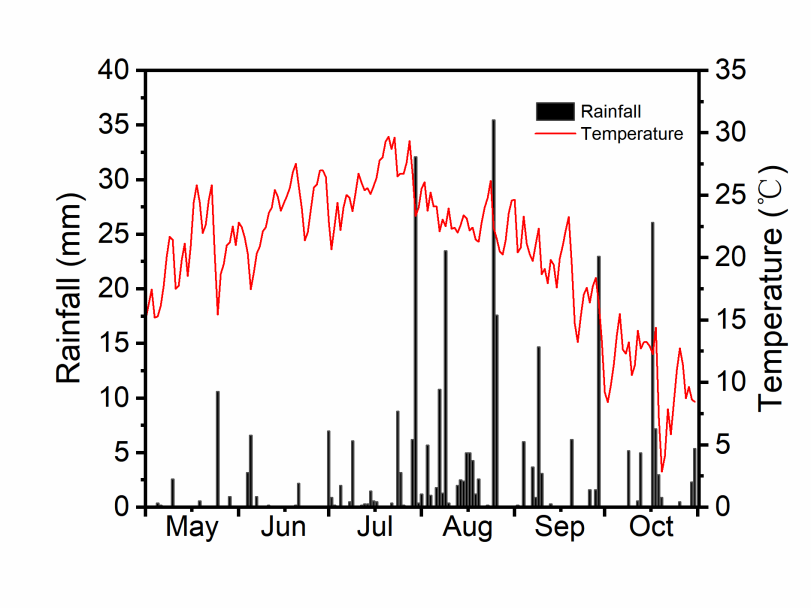


Fig S1. Daily precipitation and mean air temperature during the entire experimental period


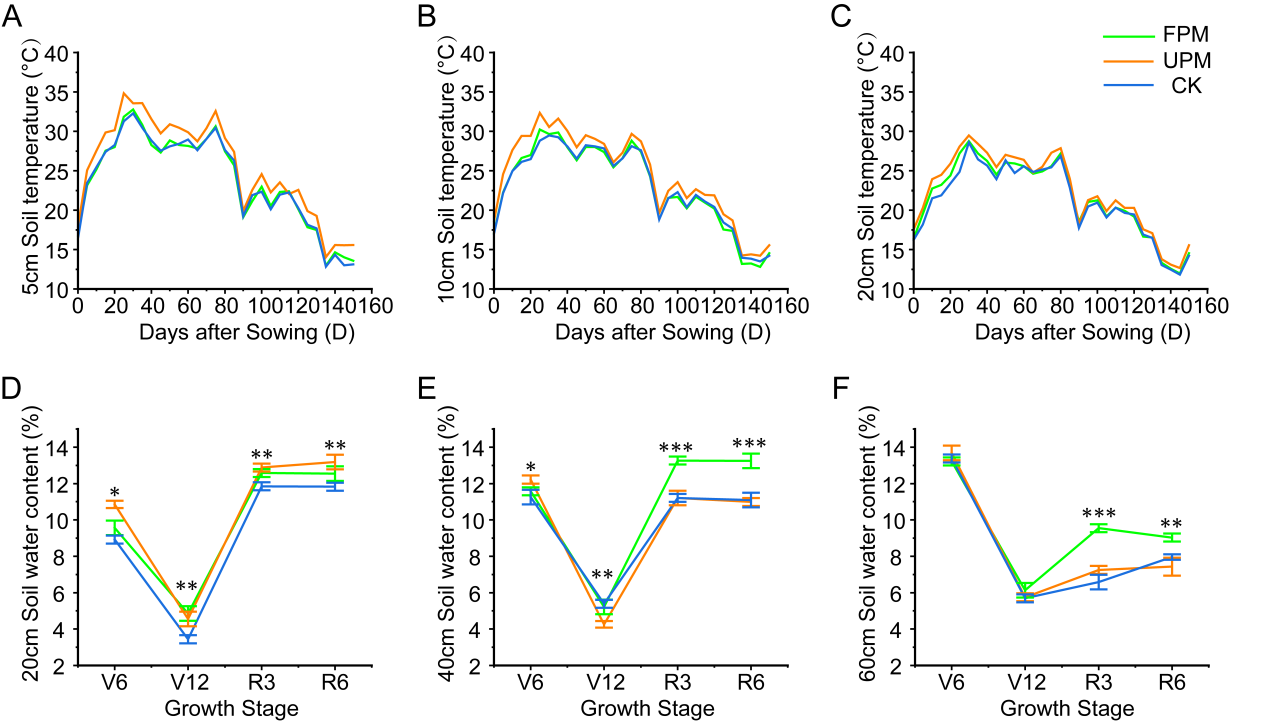


Fig S2. Effects of different mulching practices on the dynamics of soil temperature and soil water content.(A–C) Soil temperature dynamics (°C) at depths of 5, 10, and 20 cm throughout the entire growing season. The x-axis indicates days after sowing (DAS). (D–F) Soil water content (%) in the 20, 40, and 60 cm soil layers at critical developmental stages (V6, V12, R3, and R6). The lines represent the three treatments: FPM (green), UPM (orange), and CK (blue). Data in panels (D–F) are presented as the mean ± standard error (SE) (n = 3). Asterisks denote significant differences between the mulching treatments (FPM or UPM) and the CK control at each respective developmental stage (P < 0.05, *P < 0.01, **P < 0.001).


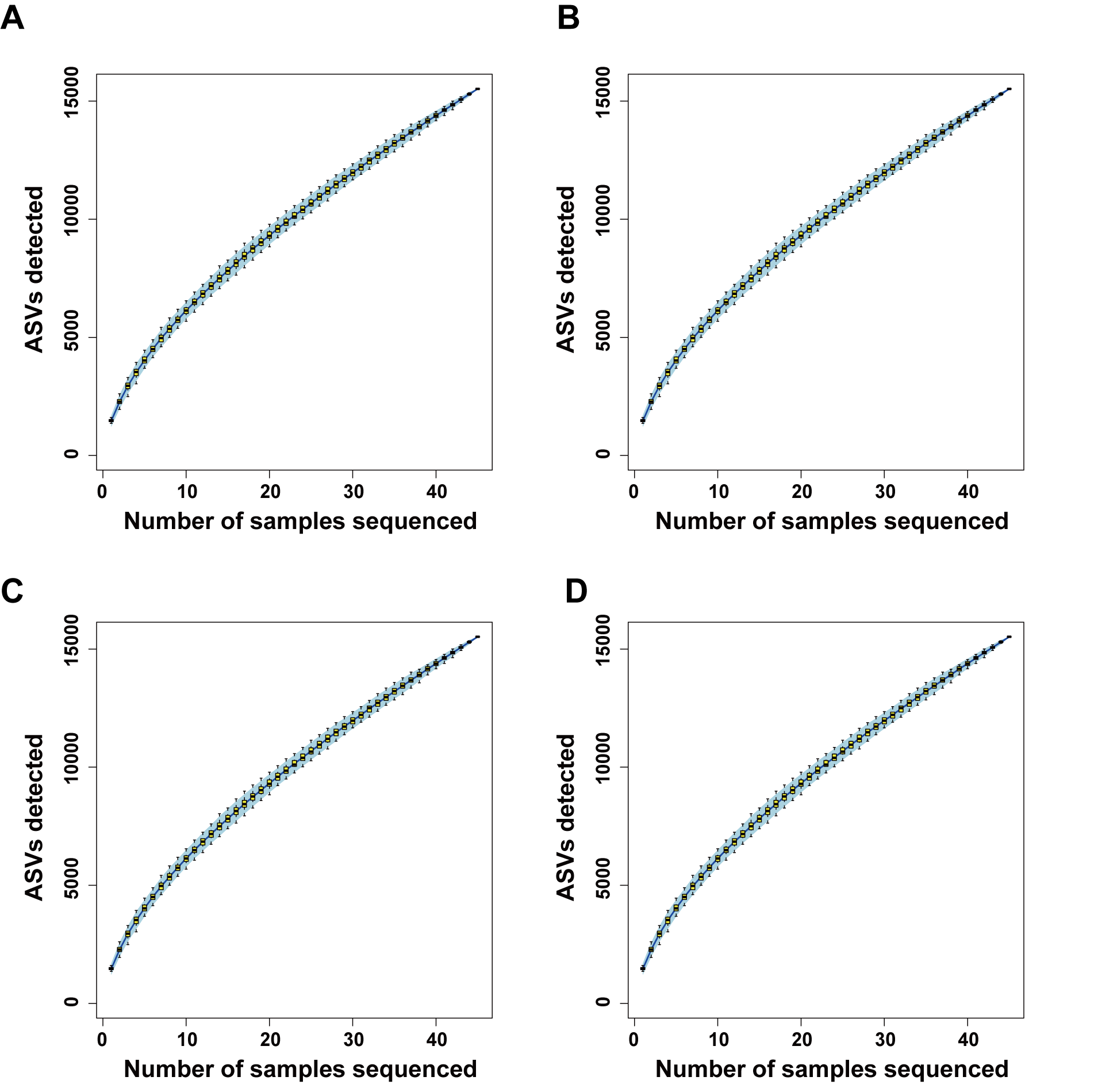


Fig S3. Species accumulation curves for bacterial and fungal communities. (A) bacteria at the V12 stage, (B) bacteria at the R6 stage, (C) fungi at the V12 stage, and (D) fungi at the R6 stage.


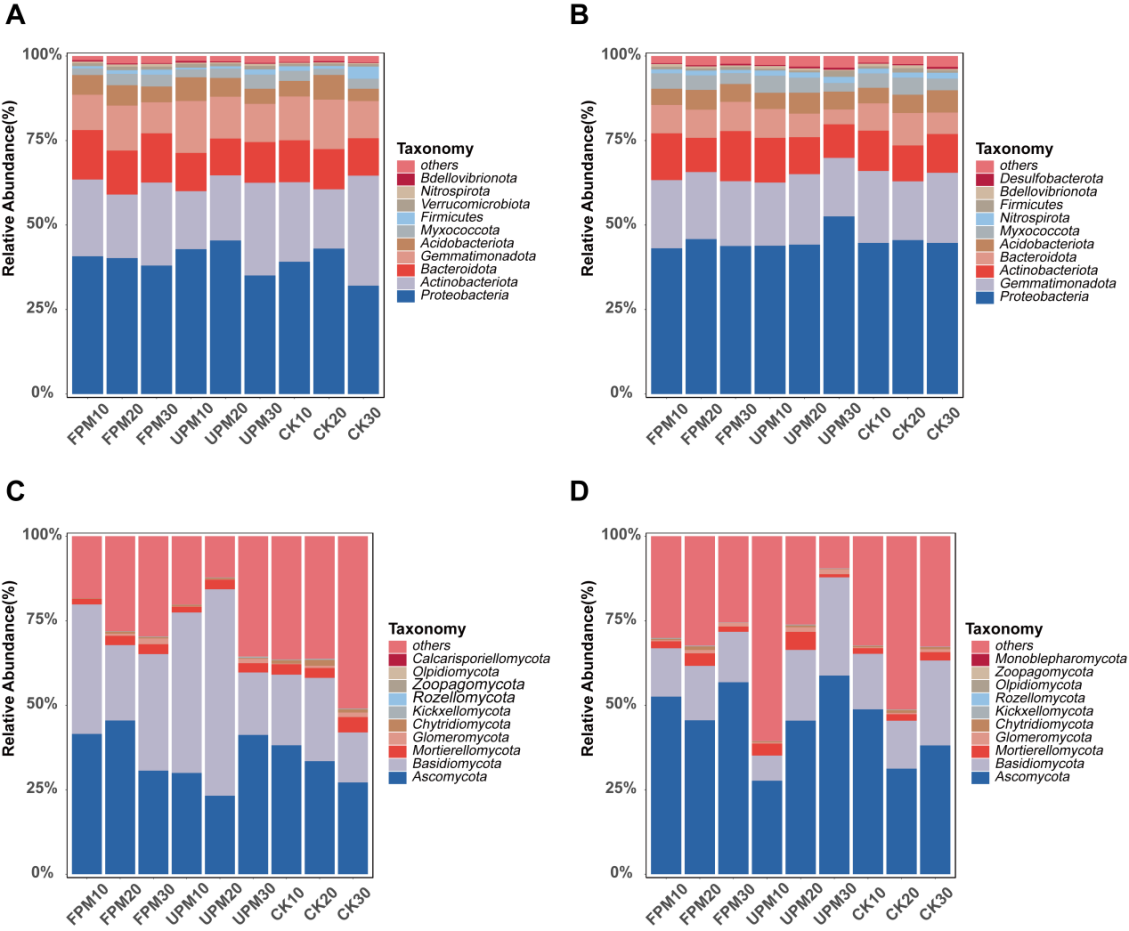


Fig S4. Relative abundance of microbial communities at the phylum level across different treatments and developmental stages.(A, B) The ten most abundant phyla in the bacterial community at the V12 and R6 developmental stages, respectively. (C, D) The ten most abundant phyla in the fungal community at the V12 and R6 developmental stages, respectively. The x-axis represents the different combinations of treatments and soil depths (10 cm, 20 cm, and 30 cm). The y-axis indicates the relative abundance (%). The legend lists the top ten most abundant phyla; all other less abundant phyla are grouped as 'Others'.


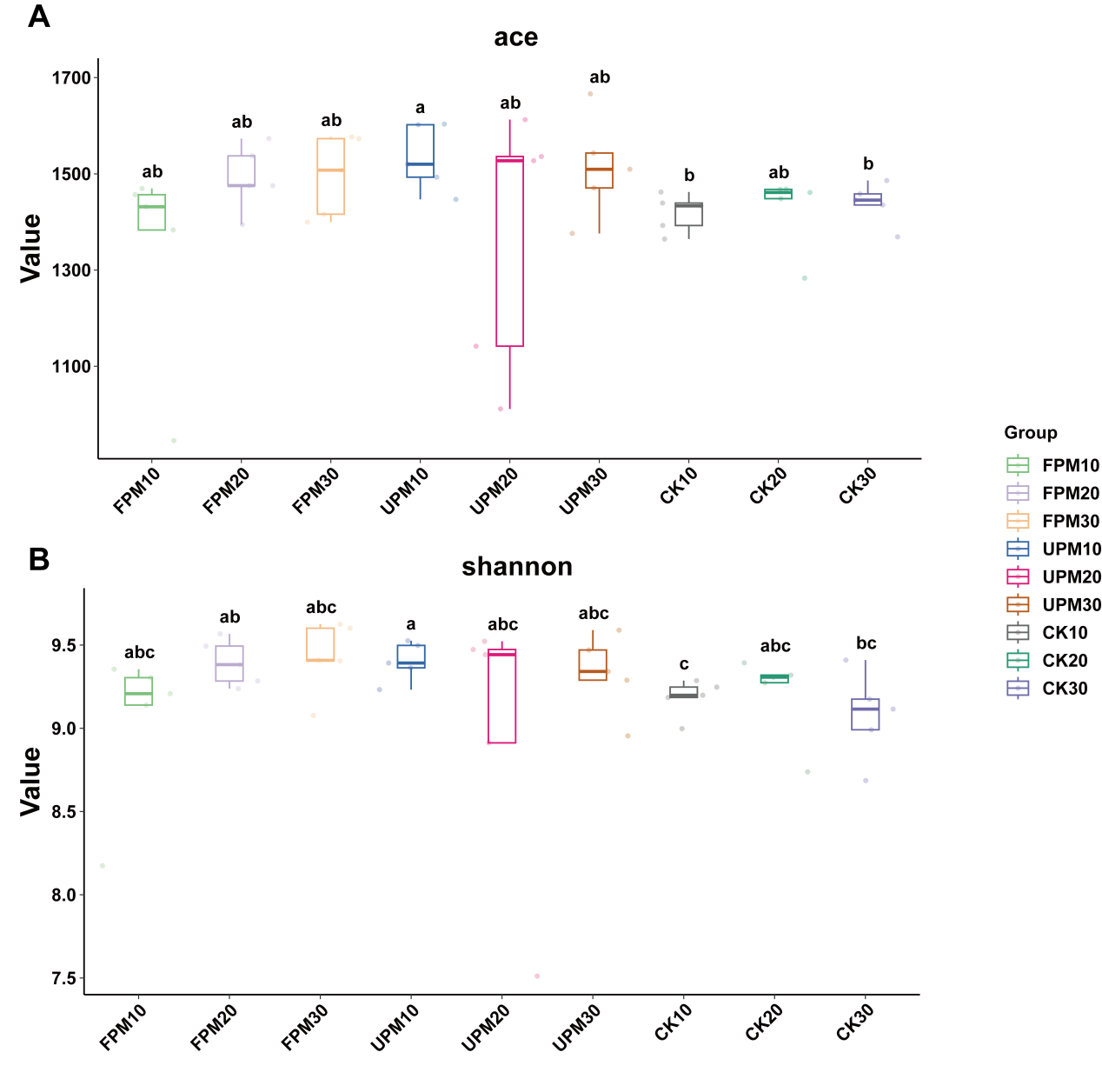


Fig S5. Alpha diversity indices of the bacterial community at the V12 stage. (A) ACE index and (B) Shannon index.


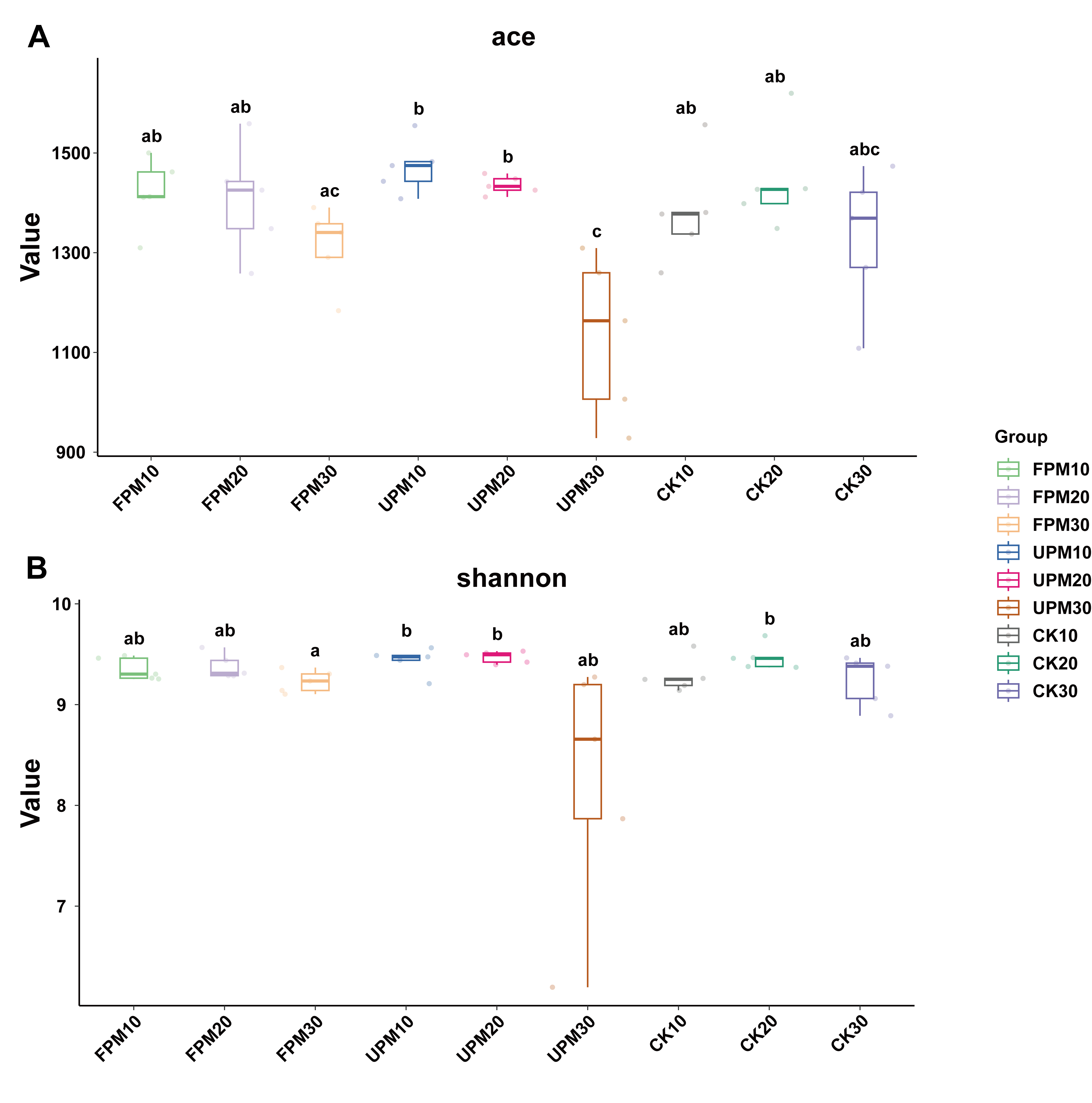


Fig S6. Alpha diversity indices of the bacterial community at the R6 stage. (A) ACE index and (B) Shannon index.


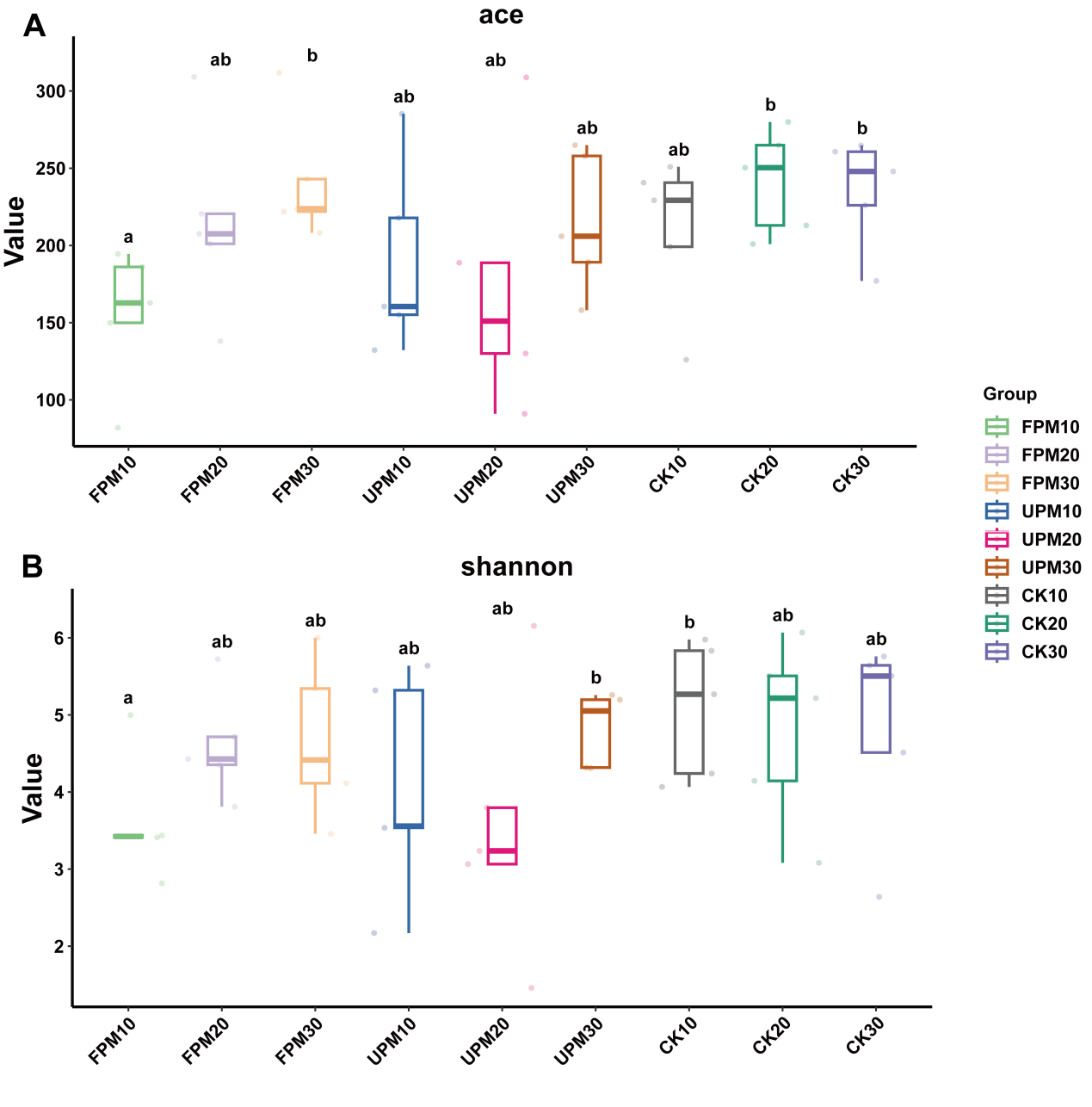


Fig S7. Alpha diversity indices of the fungal community at the V12 stage. (A) ACE index and (B) Shannon index.


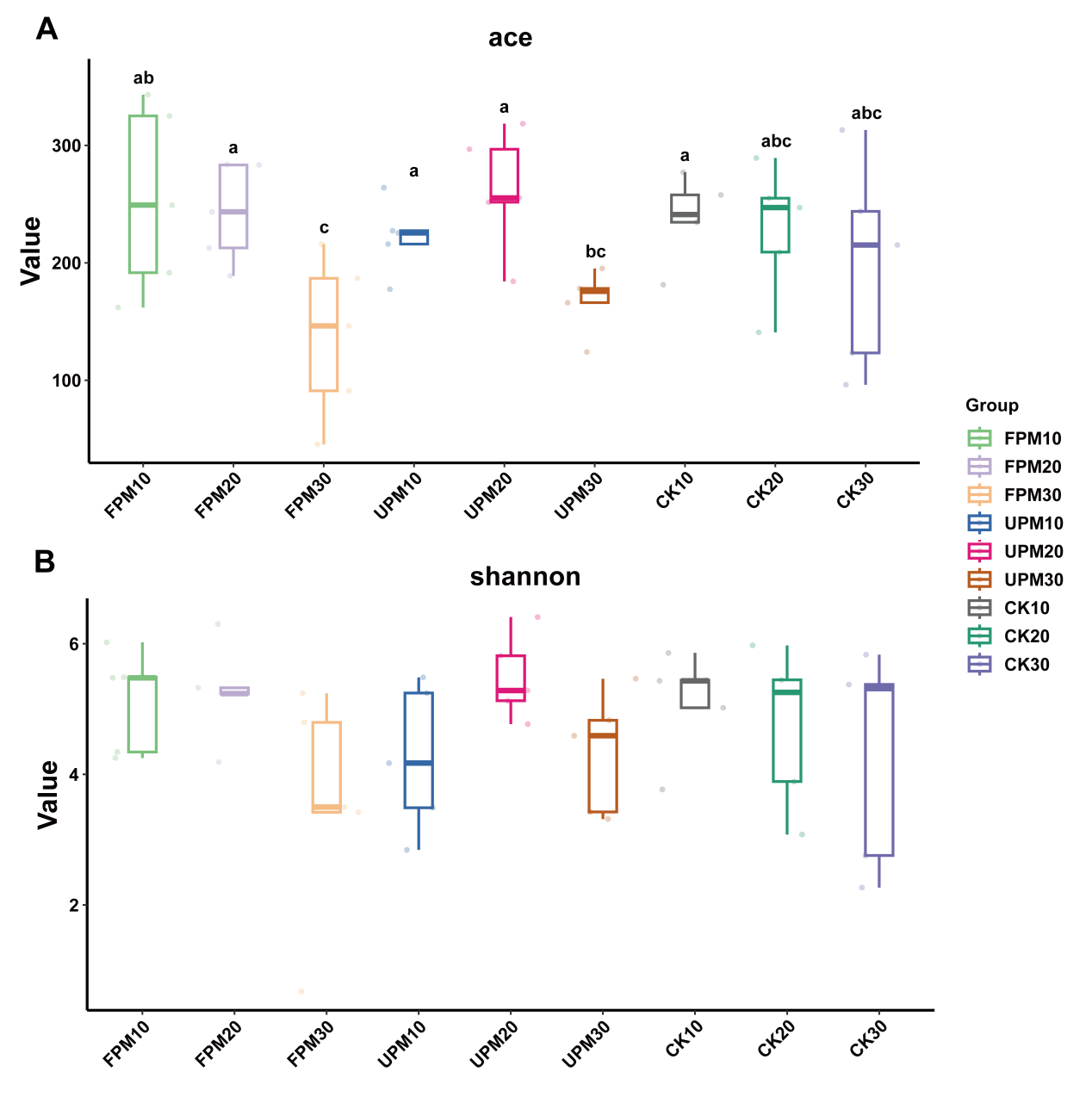


Fig S8. Alpha diversity indices of the fungal community at the R6 stage. (A) ACE index and (B) Shannon index.


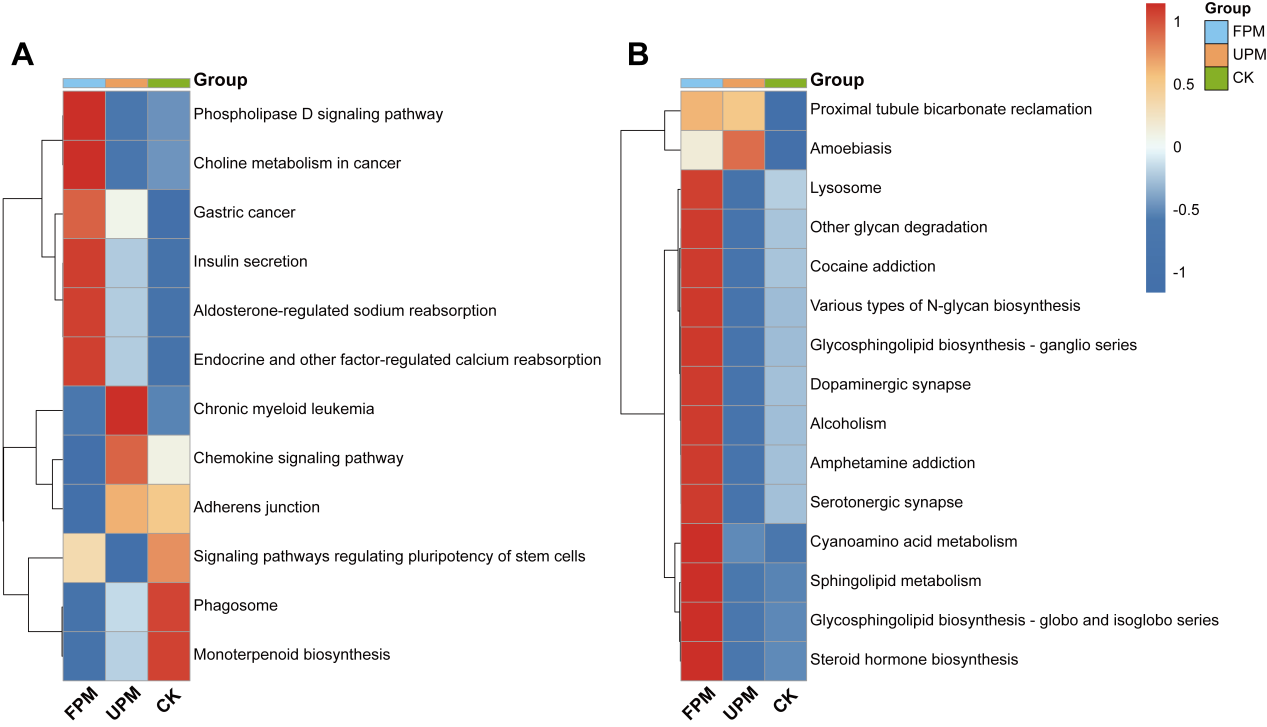


Fig S9. Top 15 differentially abundant KEGG Level 3 pathways in the bacterial community, predicted by PICRUSt2.results for (A) the V12 stage and (B) the R6 stage.


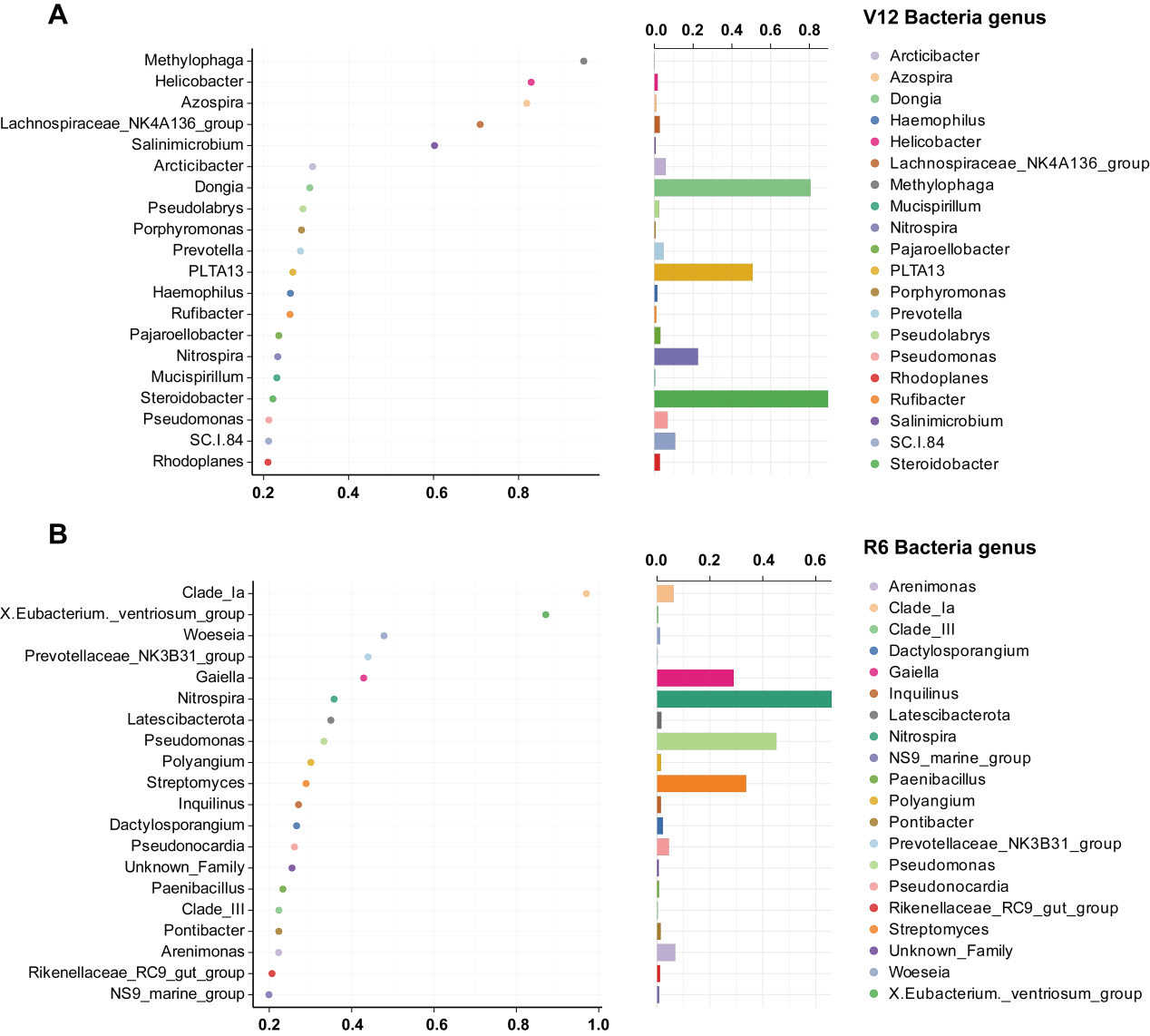


Fig S10. Random Forest model results for the bacterial community. the most important discriminant genera at (A) the V12 stage and (B) the R6 stage.


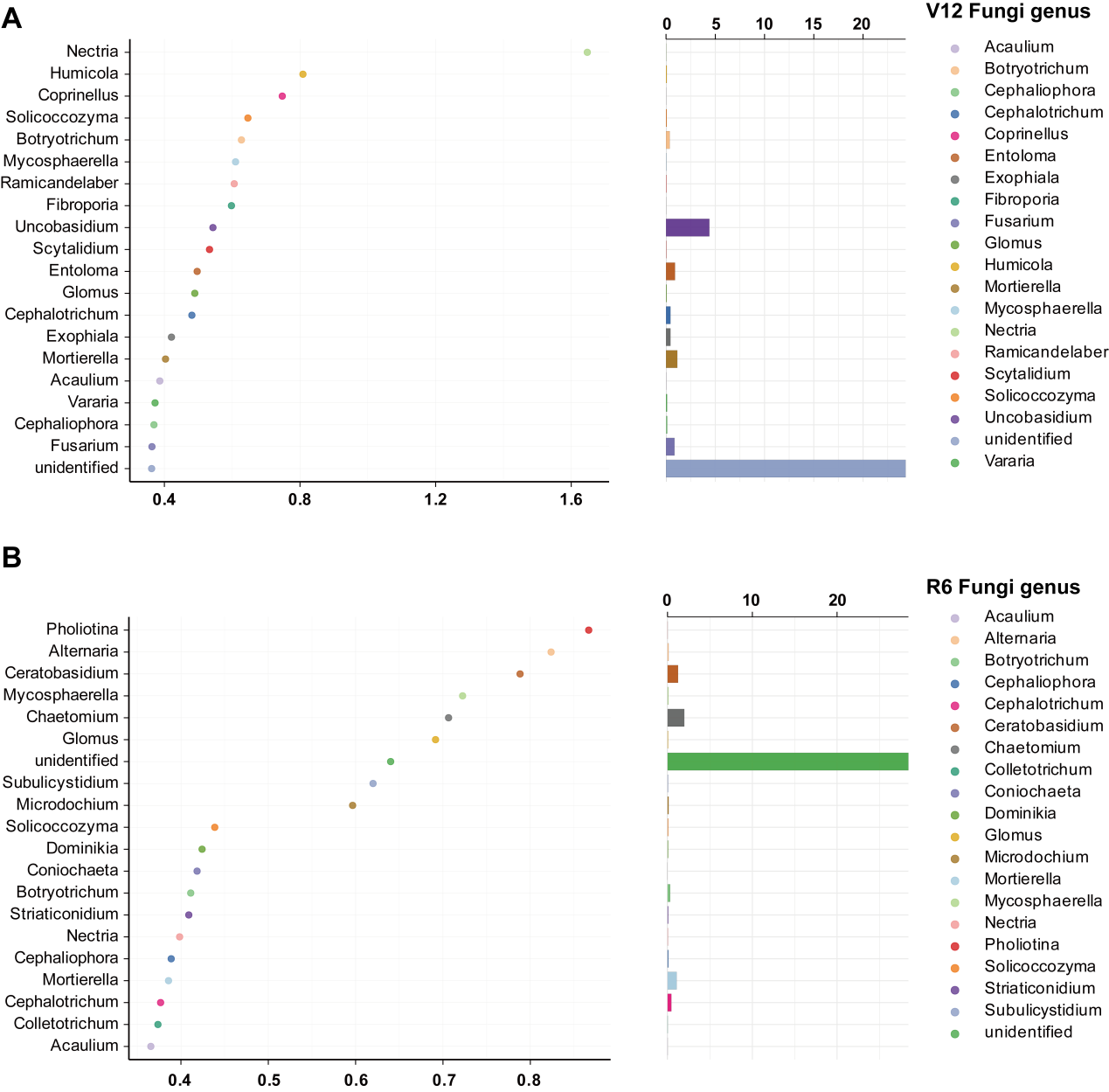


Fig S11. Random Forest model results for the fungal community. the most important discriminant genera at (A) the V12 stage and (B) the R6 stage.


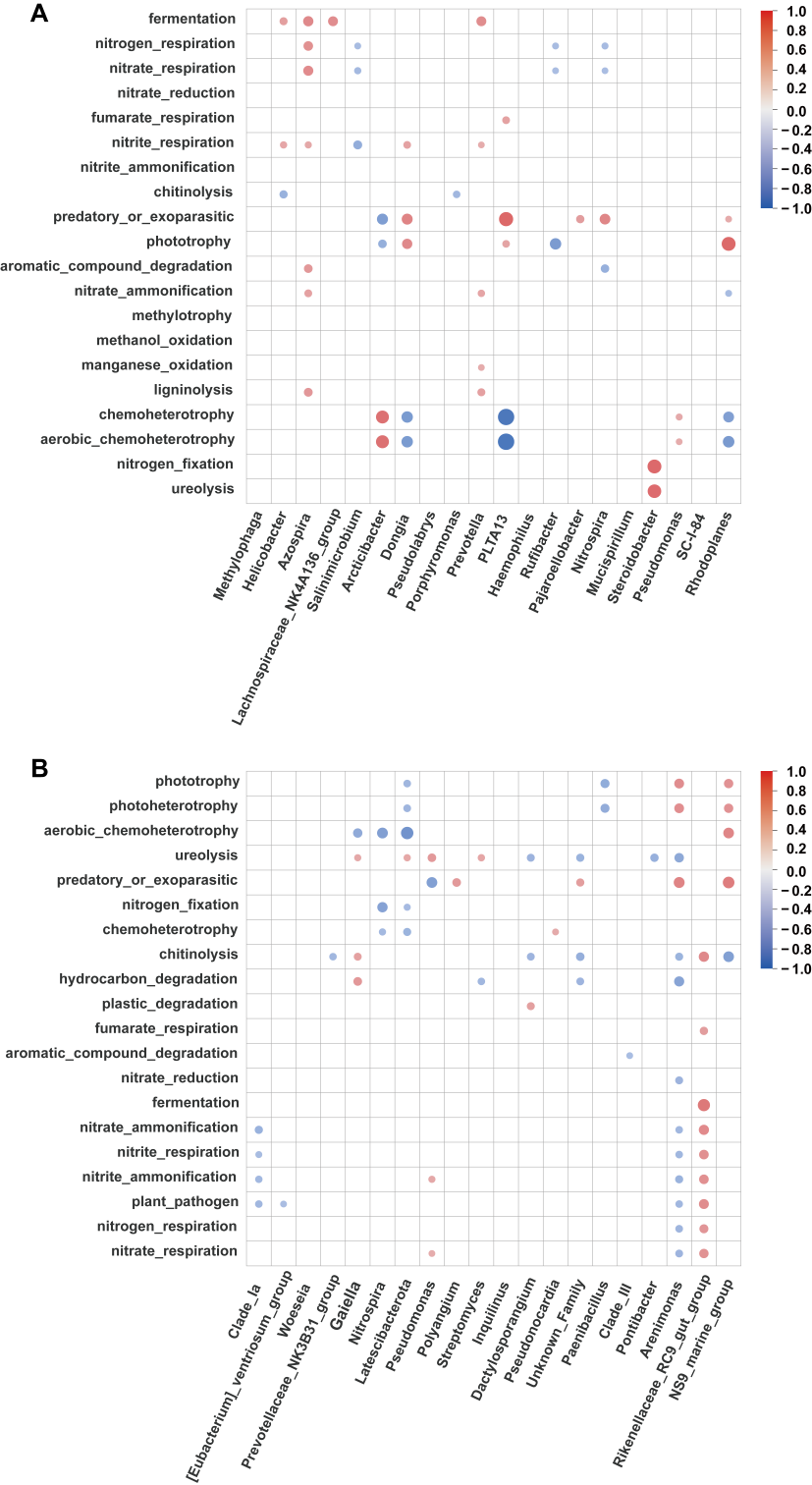


Fig S12. Spearman correlation analysis between the relative abundances of key bacterial genera (identified by random forest models) and the relative abundances of the top 20 predicted ecological functions (FAPROTAX). The analysis was conducted for the (A) V12 and (B) R6 growth stages. The color of the circles indicates the direction of the correlation (red for positive, blue for negative), and the size of the circles is proportional to the absolute value of the Spearman correlation coefficient (ρ). Only significant correlations (P < 0.05) are displayed.


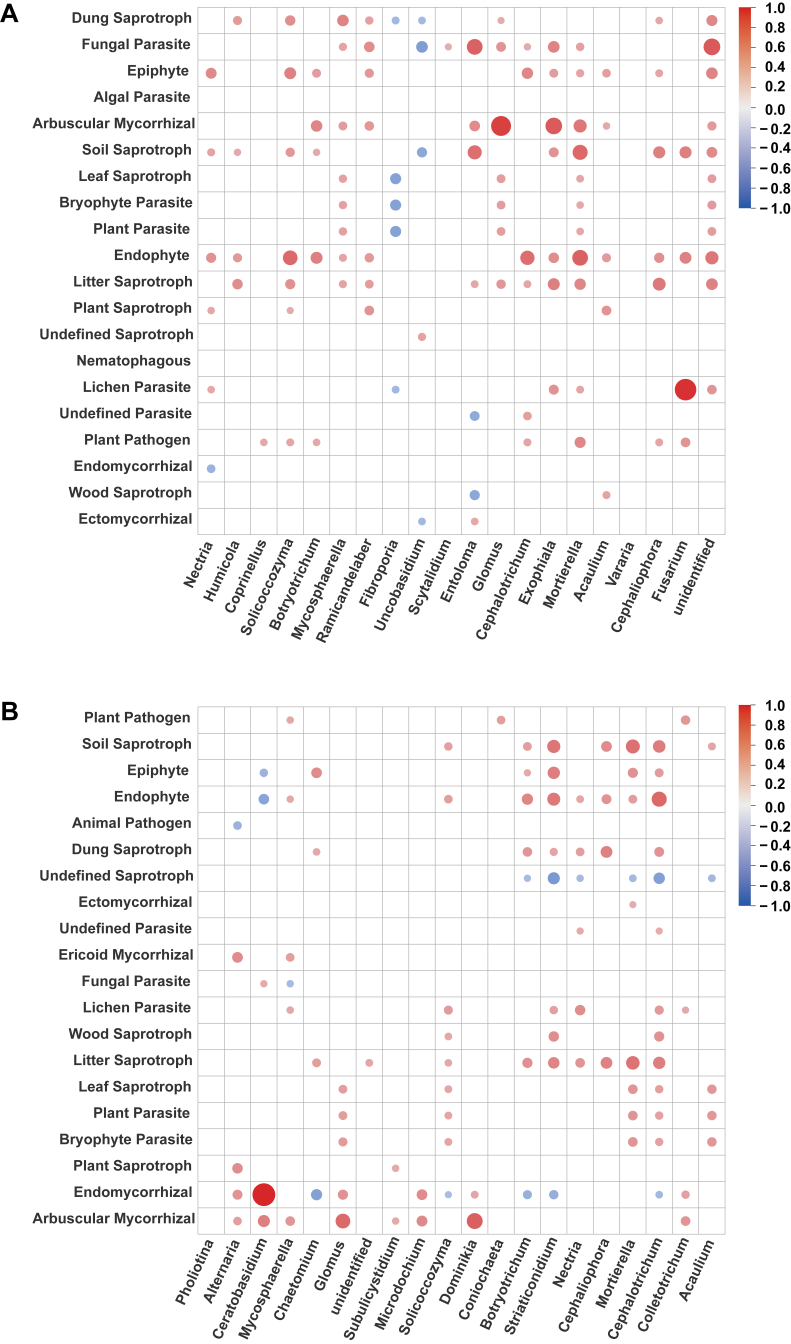


Fig S13. Spearman correlation analysis between the relative abundances of key fungal genera (identified by random forest models) and the relative abundances of predicted ecological guilds (FUNGuild). The analysis was conducted for the (A) V12 and (B) R6 growth stages. The color of the circles indicates the direction of the correlation (red for positive, blue for negative), and the size of the circles is proportional to the absolute value of the Spearman correlation coefficient (ρ). Only significant correlations (P < 0.05) are displayed.
